# Exceptionally long-lived light-emitting electrochemical cells: multiple intra-cation π-stacking interactions in [Ir(C^N)_2_(N^N)][PF_6_] emitters†
†Electronic supplementary information (ESI) available: Experimental details. Fig. S1–S10: additional structural and NMR spectroscopic figures; voltage *vs.* time plots for LEC devices and electroluminescence spectra. CCDC 1019226–1019229. For ESI and crystallographic data in CIF or other electronic format see DOI: 10.1039/c4sc03942d



**DOI:** 10.1039/c4sc03942d

**Published:** 2015-03-06

**Authors:** Andreas M. Bünzli, Edwin C. Constable, Catherine E. Housecroft, Alessandro Prescimone, Jennifer A. Zampese, Giulia Longo, Lidón Gil-Escrig, Antonio Pertegás, Enrique Ortí, Henk J. Bolink

**Affiliations:** a Department of Chemistry , University of Basel , Spitalstrasse 51 , CH4056 Basel , Switzerland . Email: edwin.constable@unibas.ch ; Email: catherine.housecroft@unibas.ch ; Tel: +41 61 267 1008; b Instituto de Ciencia Molecular , Universidad de Valencia , Catedrático José Beltrán 2 , Paterna , E-46980 , Spain . Email: henk.bolink@uv.es; c Fundació General de la Universitat de Valencia (FGUV) , PO Box 22085 , Valencia , Spain

## Abstract

A series of cyclometalated iridium(iii) complexes [Ir(C^N)_2_(N^N)][PF_6_] (N^N = 2,2′-bipyridine (**1**), 6-phenyl-2,2′-bipyridine (**2**), 4,4′-di-*tert*-butyl-2,2′-bipyridine (**3**), 4,4′-di-*tert*-butyl-6-phenyl-2,2′-bipyridine (**4**); HC^N = 2-(3-phenyl)phenylpyridine (HPhppy) or 2-(3,5-diphenyl)phenylpyridine (HPh_2_ppy)) are reported. They have been synthesized using solvento precursors so as to avoid the use of chlorido-dimer intermediates, chloride ion contaminant being detrimental to the performance of [Ir(C^N)_2_(N^N)][PF_6_] emitters in light-electrochemical cell (LEC) devices. Single crystal structure determinations and variable temperature solution ^1^H NMR spectroscopic data confirm that the pendant phenyl domains engage in multiple face-to-face π-interactions within the coordination sphere of the iridium(iii) centre. The series of [Ir(Phppy)_2_(N^N)]^+^ and [Ir(Ph_2_ppy)_2_(N^N)]^+^ complexes investigated include those with and without intra-cation face-to-face π-stacking. All the complexes display excellent luminescent properties, in particular when employed in thin solid films. The most important observation is that all the LECs using the [Ir(Phppy)_2_(N^N)]^+^ and [Ir(Ph_2_ppy)_2_(N^N)]^+^ emitters (*i.e.* with and without intra-cation π-stacking interactions) exhibit very stable luminance outputs over time, even when driven at elevated current densities. The most stable LEC had an extrapolated lifetime of more than 2500 hours under accelerated testing conditions.

## Abstract 2

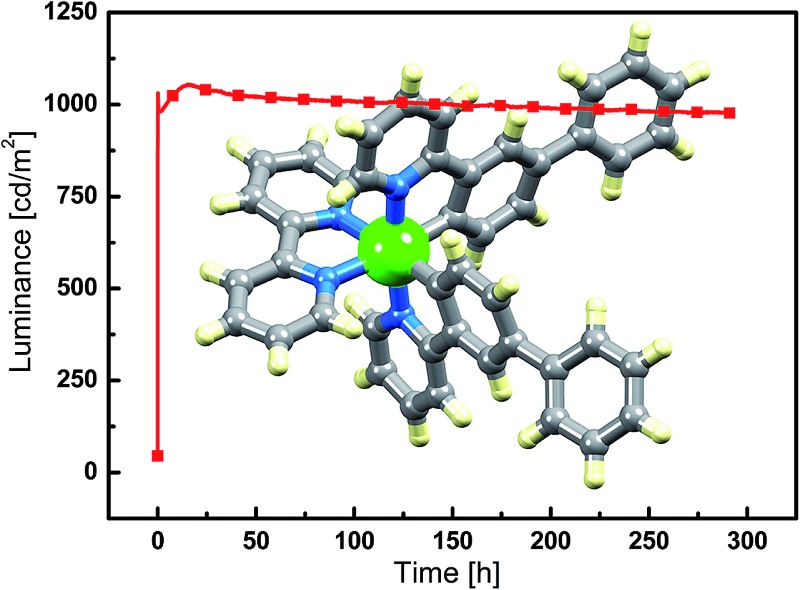
Extremely long-lived LEC devices have been achieved using [Ir(C^N)_2_(bpy)]^+^ complexes with phenyl-substituted C^N ligands.

## Introduction

Light-emitting electrochemical cells (LECs) are a class of light-emitting devices in which the active material is a charged species (an ionic transition-metal complex, iTMC–LECs, or a polymer, PLECs).^1^ LECs operate in a unique fashion: after application of a bias, the charged species in the active layer move towards the electrodes, accumulating at the interfaces and causing a sharp drop of potential near the electrode interfaces with the consequent formation of doped zones. In this situation emission of light takes place at the intrinsic region.^2^ As a consequence of this behaviour, it is not necessary for LECs to incorporate a low work-function metal, because the injection barrier of the charges is reduced by the formation of an electric double layer. Air-stable electrodes such as Al can be used, negating the need for rigorous encapsulation of the device as is essential for organic light-emitting diodes (OLEDs). Compared to OLEDs, LECs possess a simpler architecture allowing them to be prepared by solution processes.

The first report of an iTMC–LEC was by Maness *et al.*^3^ and utilized a ruthenium(ii)-containing complex as the single component in the active layer. The emission band of ruthenium(ii) complexes such as those based on [Ru(bpy)_3_]^2+^ is centred in the orange-red region, and this limits the emission colours that can be achieved with this class of compound. Even more problematic is the low stability of these materials under device conditions. By changing from a second to a third row transition metal (*e.g.* iridium) in the iTMC, it is possible to improve the stability of the device and achieve higher ligand-field splitting energies leading to higher colour tunability.^4–7^

The use of iTMCs containing ligands with substituents that are capable of intra-cation face-to-face π-interactions can stabilize the complex in the excited state and consequently enhance the lifetime of the LEC device. This strategy has been used to produce LECs with lifetimes of thousands of hours.^8–12^ The archetype member of the family is [Ir(ppy)_2_(bpy)]^+^ (Hppy = 2-phenylpyridine, bpy = 2,2′-bipyridine). Within the octahedral sphere, a 6-phenyl substituent introduced into the bpy unit is perfectly positioned to stack over the phenyl ring of the cyclometalated ppy domain. The interaction is present in both the ground and excited states of the complex, stabilizing it with respect to attack at the metal centre by nucleophiles such as H_2_O. The phenomenon was initially established with phenyl···phenyl π-interactions,^8–11^ but is also effective for other aryls, *e.g.* phenyl···pyrazolyl^12,13^ and phenyl···pyridyl contacts.^14^ Surprisingly, replacing 6-phenyl-2,2′-bipyridine (6-Phbpy) by 6,6′-diphenyl-2,2′-bipyridine (6,6′-Ph_2_bpy) does not result in additional enhancement of LEC device lifetimes on going from [Ir(ppy)_2_(6-Phbpy)]^+^ to [Ir(ppy)_2_(6,6′-Ph_2_bpy)]^+^.^8^

We now report a series of new [Ir(C^N)_2_(N^N)]^+^ complexes in which both the C^N and N^N domains contain pendant phenyl substituents and demonstrate the effects of differing degrees of π-stacking interactions in the coordination sphere of the iridium(iii) centre on the emission behaviours and LEC device characteristics. In addition, we use a solvento-iridium(iii) precursor to circumvent the detrimental effects associated with chlorido-impurities.^15^ When used as the primary active component, these complexes lead to LECs with exceptional stabilities.

## Experimental

All experimental details including crystallographic data and device preparation are given in the ESI.†

## Results and discussion

### Solvento-precursors [Ir(C^N)_2_(MeOH)_2_][PF_6_]

**Scheme 1:**
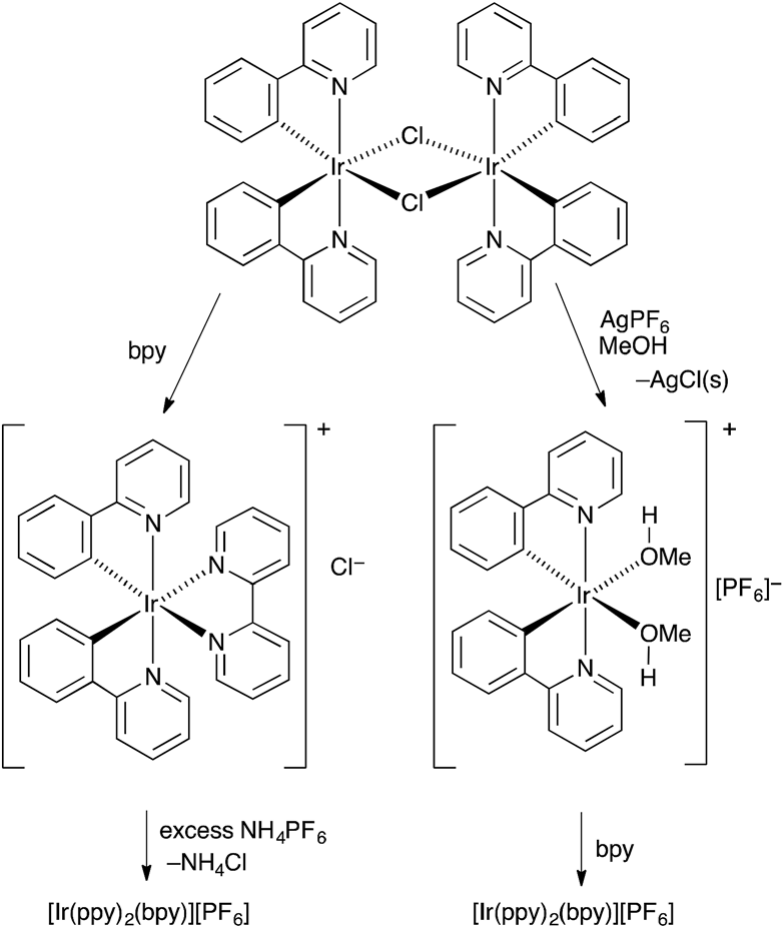
Alternative routes from [Ir_2_(ppy)_4_Cl_2_] to [Ir(ppy)_2_(bpy)][PF_6_].

The conventional method for preparing [Ir(C^N)_2_(N^N)][PF_6_] compounds is to treat the chlorido dimer [Ir_2_(C^N)_4_Cl_2_] with two equivalents of an N^N chelating ligand, followed by anion exchange by addition of NH_4_PF_6_ (Scheme 1, left).^16^ However, even small amounts of residual chloride ion in the final product result in significant reductions in the performance of the iTMC in LECs.^15^ A chloride-free route to [Ir(C^N)_2_(N^N)][PF_6_] salts is highly desirable, although the use of commercial iridium chloride starting materials such as IrCl_3_·*x*H_2_O and Na_3_[IrCl_6_] is often hard to avoid. Solvento-complexes of the type described by Watts and coworkers^17^ appeared to be a viable alternative, and we have established a route to [Ir(C^N)_2_(N^N)][PF_6_] *via* the intermediate complexes [Ir(C^N)_2_(MeOH)_2_][PF_6_]. This is illustrated in Scheme 1 (right) with [Ir_2_(ppy)_4_Cl_2_]). The coordinated solvent must be sufficiently labile to allow displacement with an N^N ligand in the final step. Use of AgPF_6_ results in precipitation of chloride ion as AgCl, and we have previously shown that this is a reliable means of removing residual Cl^–^ from [Ir(ppy)_2_(bpy)][PF_6_] to give material with optimal LEC performance.^15^

**Scheme 2:**
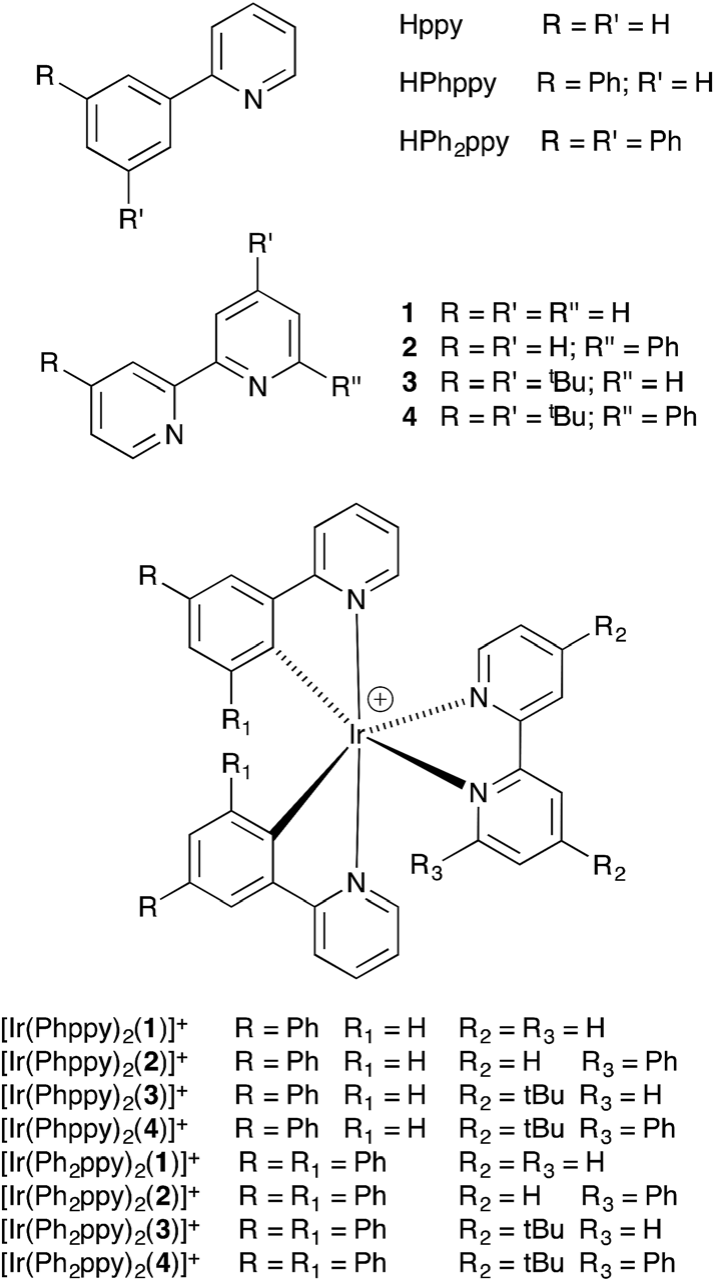
Ligand structures and abbreviations, and iridium(iii) complexes.

The C^N and N^N ligands used in this study are shown in Scheme 2, and the single crystal structure of HPh_2_ppy is described in the ESI (Fig. S1†). Reactions of [Ir_2_(Phppy)_4_Cl_2_] or [Ir_2_(Ph_2_ppy)_4_Cl_2_] (prepared using a standard method)^18^ with AgPF_6_ in MeOH resulted in the quantitative formation of [Ir(Phppy)_2_(MeOH)_2_][PF_6_] and [Ir(Ph_2_ppy)_2_(MeOH)_2_][PF_6_]. ^1^H and ^13^C NMR spectra of CD_3_OD solutions of the complexes were consistent with the formulations. Most importantly, for each compound, a singlet at *δ* 3.35 ppm in the ^1^H NMR spectrum correlating in the HMQC spectrum with a signal at *δ* 49.9 ppm was assigned to the coordinated MeOH; the proton resonance was distinct from the multiplet arising from residual bulk CD_2_HOD (Fig. S2†). Attempts to obtain electrospray mass spectrometric evidence for the [Ir(Phppy)_2_(MeOH)_2_]^+^ or [Ir(Ph_2_ppy)_2_(MeOH)_2_]^+^ ions were not successful, presumably because of the lability of the methanol molecules. In the spectrum of [Ir(Ph_2_ppy)_2_(MeOH)_2_][PF_6_], a peak envelope at *m*/*z* 805.5 corresponding to [Ir(Ph_2_ppy)_2_]^+^ was observed; the isotope pattern matched that calculated. The solvento-complexes were used in the subsequent steps as soon after synthesis as possible.

### Synthesis [Ir(C^N)_2_(N^N)][PF_6_] and structural determinations

The complexes [Ir(Phppy)_2_(N^N)][PF_6_] and [Ir(Ph_2_ppy)_2_(N^N)][PF_6_] with N^N = **1–4** (Scheme 2) were prepared by reaction of [Ir(Phppy)_2_(MeOH)_2_][PF_6_] or [Ir(Ph_2_ppy)_2_(MeOH)_2_][PF_6_] with the N^N ligand in MeOH at room temperature. The base peak in the electrospray mass spectrum of each compound corresponded to [M – PF_6_]^+^.

**Fig. 1:**
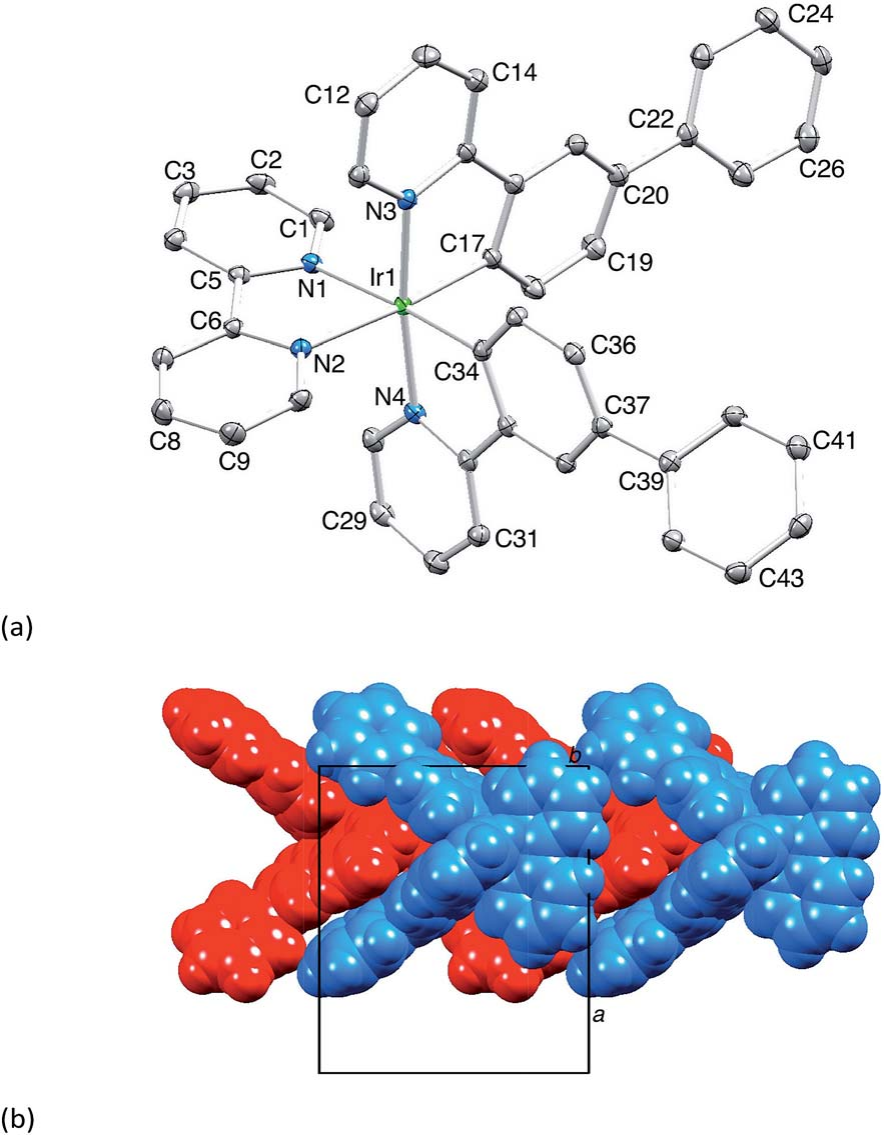
(a) Structure of the Δ-[Ir(Phppy)_2_(**1**)]^+^ cation in racemic [Ir(Phppy)_2_(**1**)][PF_6_] (H atoms omitted, ellipsoids plotted at 40% probability). Selected bond parameters: Ir1–C17 = 2.010(2), Ir1–C34 = 2.011(2), Ir1–N4 = 2.0442(18), Ir1–N3 = 2.0517(17), Ir1–N2 = 2.1349(18), Ir1–N1 = 2.1399(18) Å; N2–Ir1–N1 = 76.56(7), C17–Ir1–N3 = 80.46(8), C34–Ir1–N4 = 80.60(8)°. (b) Tight embraces of arene domains between cations.

**Fig. 2:**
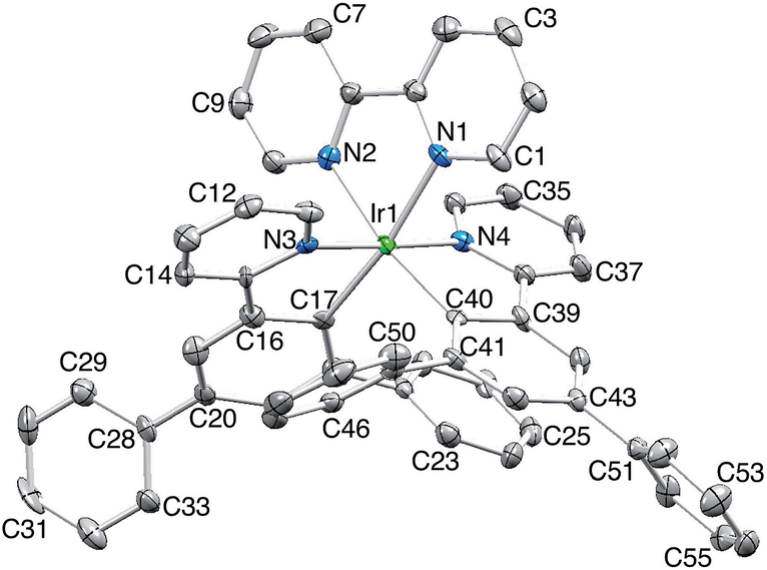
The structure of the Λ-[Ir(Ph_2_ppy)_2_(**1**)]^+^ cation in racemic [Ir(Ph_2_ppy)_2_(**1**)][PF_6_]·EtOH (H atoms omitted, ellipsoids plotted at 40% probability). Selected bond parameters: Ir1–C17 = 2.027(9), Ir1–N3 = 2.043(9), Ir1–C40 = 2.052(9), Ir1–N4 = 2.078(8), Ir1–N1 = 2.125(8), Ir1–N2 = 2.152(7) Å; N1–Ir1–N2 = 76.7(3), C17–Ir1–N3 = 80.9(3), C40–Ir1–N4 = 81.5(3)°.

**Fig. 3:**
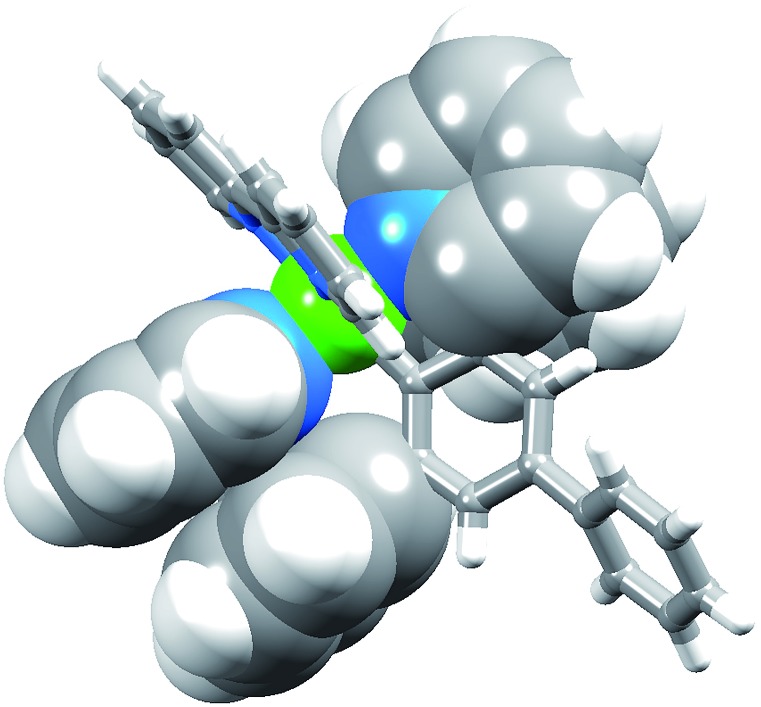
Face-to-face phenyl···pyridine π-interactions in [Ir(Ph_2_ppy)_2_(**1**)]^+^.

[Ir(Phppy)_2_(**1**)][PF_6_] and [Ir(Ph_2_ppy)_2_(**1**)][PF_6_]·EtOH crystallize in the monoclinic space group *P*2_1_/*n* and orthorhombic space group *Pna*2_1_, respectively, each with one cation in the asymmetric unit (Fig. 1a and 2). The octahedral iridium(iii) tris-chelates are chiral and in both structures, the Λ and Δ-enantiomers are present in the lattice. The bpy unit in **1** is slightly twisted in [Ir(Phppy)_2_(**1**)]^+^ (angle between the bpy ring planes = 13.1°) but is close to planar in [Ir(Ph_2_ppy)_2_(**1**)]^+^ (angle = 6.3°). In [Ir(Phppy)_2_(**1**)]^+^, both ppy units are close to planar (angles between the planes of rings containing N4/C34 and N3/C17 = 5.1 and 4.1°, respectively); the corresponding angles in the [Ir(Ph_2_ppy)_2_(**1**)]^+^ cation are 4.4 and 12.6°. The pendant phenyl substituents in [Ir(Phppy)_2_(**1**)]^+^ are twisted through 17.0 and 42.4° with respect to the cyclometalated ring to which they are bonded, and the corresponding angles in [Ir(Ph_2_ppy)_2_(**1**)]^+^ are 46.2 and 46.8°. The additional phenyl rings in [Ir(Ph_2_ppy)_2_(**1**)]^+^ (those containing C23 and C46, Fig. 2) are twisted through 75.4 and 61.8°, respectively, and these large twist angles are associated with face-to-face π-stacking of these rings over the [Ph_2_ppy]^–^ pyridine rings containing N3 and N4 (Fig. 3). The π-interaction between the rings containing N4 and C23 is characterized by an angle between ring planes of 9.9°, phenyl ring plane···centroid of pyridine ring distance of 3.27 Å, and centroid···centroid separation of 3.48 Å. The corresponding parameters for the π-stacking of rings with N3 and C46 are 18.6°, 3.37 Å and 3.51 Å. The cations in [Ir(Phppy)_2_(**1**)][PF_6_] are closely associated through embraces of the arene domains (Fig. 1b) leading to assembly of anion-separated columns running along the *b*-axis.

**Fig. 4:**
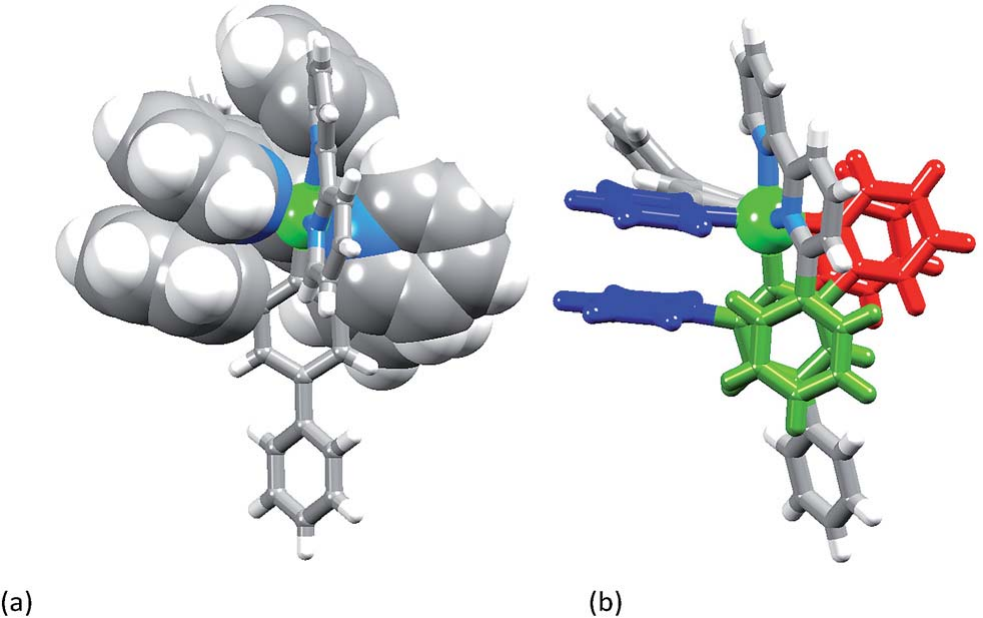
The three intra-cation face-to-face π-stacking interactions in [Ir(Ph_2_ppy)_2_(**2**)]^+^: (a) in space-filling representations, and (b) showing the two pyridyl···pendant phenyl interactions in red and blue, and the cyclometalated phenyl···pendant phenyl interaction in green.

[Ir(Ph_2_ppy)_2_(**2**)][PF_6_]·2C_6_H_5_Me crystallizes in the triclinic space group *P*1, and Fig. S3† shows the Λ-enantiomer of [Ir(Ph_2_ppy)_2_(**2**)]^+^; both enantiomers are present in the lattice. Although the three chelate angles in [Ir(Ph_2_ppy)_2_(**2**)]^+^ are comparable with those in [Ir(Phppy)_2_(**1**)]^+^ and [Ir(Ph_2_ppy)_2_(**1**)]^+^, the remaining angles in the coordination environment sphere of Ir1 vary greatly (Table 1). The widening of the *cis*-C–Ir–N angles in [Ir(Ph_2_ppy)_2_(**2**)]^+^ is coupled to the three intra-cation π-stacking interactions shown in Fig. 4. The face-to-face contacts are between pairs of phenyl and pyridine rings containing C20/N44 and C49/N2 (see Fig. S3†) and between the cyclometalated ring with C47 and pendant phenyl ring containing C38; the π-interactions are characterized by centroid···ring-plane and centroid···centroid distances and interplane angle of 3.37 Å, 3.61 Å and 14.9° between rings with C20/N44, 3.18 Å, 3.47 Å and 5.8° for rings with C49/N2, and 3.24 Å, 3.42 Å and 10.9° for rings with C47/C38. Packing interactions involve extensive CH···F contacts between cations and anions, and one of the toluene molecules engages in edge-to-face π-contacts with a pendant phenyl ring of the cation.

**Table 1:** Comparison of the non-chelate angles in the octahedral coordination sphere of Ir1 in [Ir(Phppy)_2_(**1**)]^+^, [Ir(Ph_2_ppy)_2_(**1**)]^+^ and [Ir(Ph_2_ppy)_2_(**2**)]^+^, see Fig. 1, 2 and S3†

Angles	[Ir(Phppy)_2_(**1**)]^+^	[Ir(Ph_2_ppy)_2_(**1**)]^+^	[Ir(Ph_2_ppy)_2_(**2**)]^+^
*trans*-N–Ir–N	172.08(7)	175.9(3)	174.85(7)
*trans*-N–Ir–C	175.22(7)	170.9(4)	174.64(8)
	173.96(7)	169.6(3)	169.36(7)
*cis*-N–Ir–N	88.66(7)	84.3(3)	83.03(7)
	98.37(7)	95.8(3)	95.77(7)
	98.36(7)	92.0(3)	94.32(7)
	86.89(7)	84.9(3)	80.53(7)
*cis*-C–Ir–C	87.28(8)	94.5(3)	83.91(8)
*cis*-C–Ir–N	92.71(8)	94.4(3)	93.87(8)
	94.91(8)	94.5(3)	101.37(8)
	97.46(8)	97.7(3)	104.46(8)
	98.71(7)	102.4(3)	106.35(7)

### Solution behaviour of [Ir(C^N)_2_(N^N)][PF_6_]: NMR spectroscopy

**Scheme 3:**
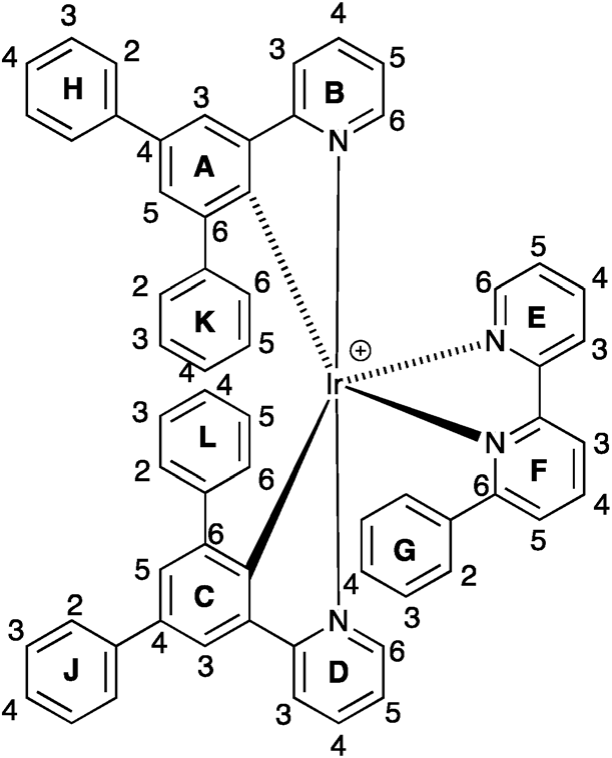
Ring and atom labelling in [Ir(Ph_2_ppy)_2_(**2**)]^+^. Analogous ring labelling is used in all complex cations.

**Fig. 5:**
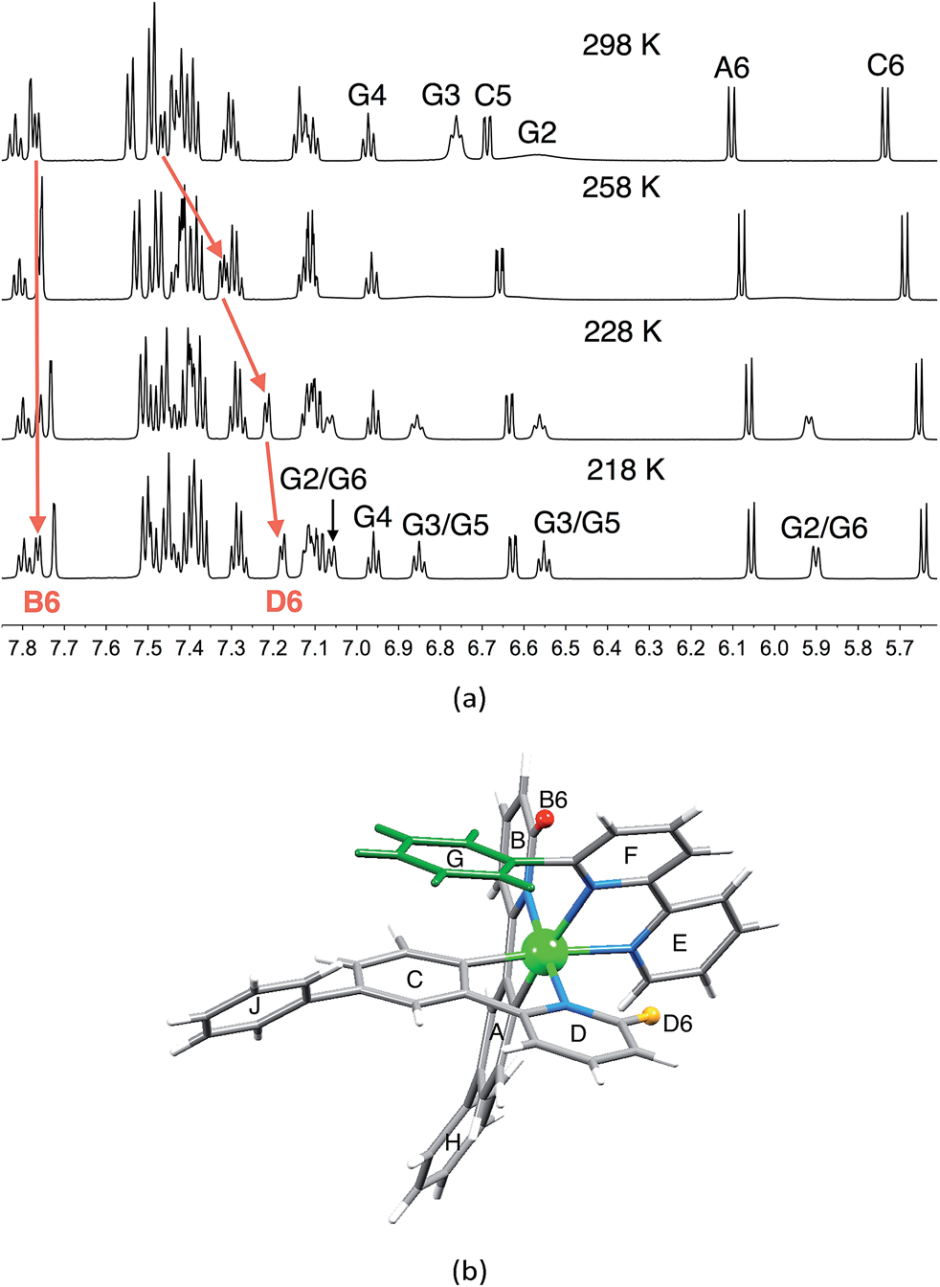
(a) Part of the 600 MHz ^1^H variable temperature NMR spectra of a CD_2_Cl_2_ solution of [Ir(Phppy)_2_(**2**)][PF_6_]; the full spectrum is shown in Fig. S5.† (b) Modelled structure of [Ir(Phppy)_2_(**2**)]^+^ with protons H^B6^ and H^D6^ and the phenyl (G) ring highlighted.

^1^H and ^13^C NMR spectra of the complexes were assigned using 2D methods; a common ring labelling scheme has been used and is shown for the most ring-rich cation in Scheme 3. For N^N = **1** or **3**, the cation is *C*_2_-symmetric (*i.e.* ring A = C, *etc.*), but for N^N = **2** or **4**, phenyl ring G desymmetrizes the structure. Fig. S4† compares the room temperature ^1^H NMR spectra of [Ir(Phppy)_2_(**1**)][PF_6_] and [Ir(Phppy)_2_(**2**)][PF_6_]. For [Ir(Phppy)_2_(**2**)][PF_6_], a broad signal at *δ* 6.57 ppm and a broadened triplet at *δ* 6.76 ppm indicate hindered rotation of phenyl ring G on the NMR timescale.^19,20^ Upon cooling (Fig. S5† and 5), the broad signals collapse and give rise at 218 K to two doublets (*δ* 5.90 and 7.06 ppm, H^G2^ and H^G6^) and two multiplets (*δ* 6.85 and 6.55 ppm, H^G3^ and H^G5^) (Fig. 5a). To understand the observations, we consider the modelled structure of [Ir(Phppy)_2_(**2**)]^+^ (Fig. 5b). Apart from H^G2/G3/G5/G6^, one other signal is significantly affected by temperature, and shifts from ≈*δ* 7.4 ppm at 298 K to *δ* 7.18 ppm at 218 K. In the HMQC spectrum at 218 K, this proton correlates to a ^13^C NMR signal at *δ* 149.4 ppm. A second high-frequency signal at *δ* 150.0 ppm correlates to a ^1^H NMR signal at *δ* 7.76 ppm which is temperature independent (Fig. 5a). The high-frequency ^13^C NMR signals are characteristic of pyridine C^6^ nuclei and are identified as C^D6^ and C^B6^; the remaining pyridine C^6^ (C^E6^) is observed at *δ* 151.2 ppm at 218 K. Protons H^B6^ or H^D6^ (red and orange in Fig. 5b) were distinguished from NOESY spectra (at 298 and 218 K). Ring G (green in Fig. 5b) is spatially closer to phenyl ring J than the corresponding phenyl ring H, and NOESY cross peaks are observed between H^G3^/H^J2^ and H^G4^/H^J2^ at 298 K, and between H^G3^/H^J2^, H^G4^/H^J2^ and H^G5^/H^J2^ at 218 K. This allows the spin systems of the two [Phppy]^–^ ligands to be discriminated. Although proton H^B6^ is closer to ring G, it is H^D6^ that is affected more as the hindered rotation of ring G is frozen out. We propose that as the π-interaction between rings C and G strengthens at low temperature, a concomitant deformation of the bpy domain (rings E and F) occurs leading to an enhanced C–H···π interaction between H^D6^ and ring E (and an associated shift to lower frequency for H^D6^). Twisting of the bpy unit is substantiated by the structural data (see above) and is also responsible for the dynamic behaviour of [Ir(ppy)_2_(Naphbpy)]^+^ (Naphbpy = 6-(2-naphthyl)-2,2′-bipyridine).^21^

While phenyl ring H in the coordinated [Phppy]^–^ ligand is free to rotate on the NMR timescale, spectroscopic data show that phenyl ring K in metal-bound [Ph_2_ppy]^–^ is static at 295 K. The data in Table S1,† and in particular the shift to lower frequency for all ring B protons on going from [Ir(Phppy)_2_(**1**)][PF_6_] to [Ir(Ph_2_ppy)_2_(**1**)][PF_6_], and from [Ir(Phppy)_2_(**3**)][PF_6_] to [Ir(Ph_2_ppy)_2_(**3**)][PF_6_], are consistent with π-stacking of rings B and K in solution, in agreement with the solid state structures (Fig. S6†).

**Fig. 6:**
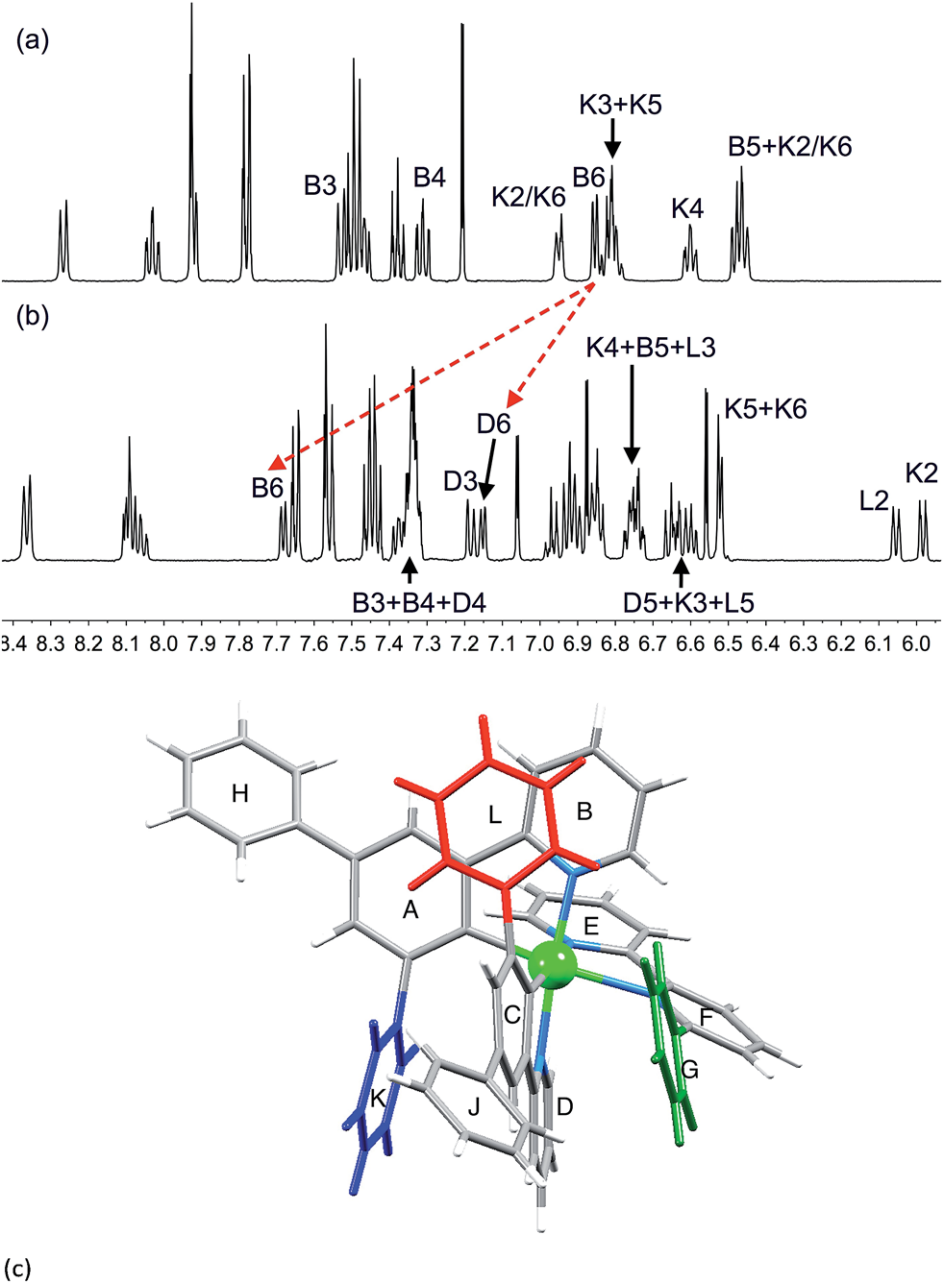
500 MHz ^1^H NMR spectrum (295 K, CD_2_Cl_2_) of (a) [Ir(Ph_2_ppy)_2_(**1**)][PF_6_] and (b) [Ir(Ph_2_ppy)_2_(**2**)][PF_6_]. Chemical shifts in *δ*/ppm. (c) Structure of [Ir(Ph_2_ppy)_2_(**2**)]^+^ (from the solid-state determination) showing rings G, K and L.

The effects of introducing a third phenyl group are seen by comparing the ^1^H NMR spectra of [Ir(Ph_2_ppy)_2_(**1**)][PF_6_] and [Ir(Ph_2_ppy)_2_(**2**)][PF_6_] (Fig. 6). Pendant rings K and L are static in [Ir(Ph_2_ppy)_2_(**2**)]^+^; each is π-stacked over an adjacent cyclometalated ligand (Fig. 6c), as indicated by the relatively low frequency shifts for signals in the B, D, K and L rings. The exceptions are the signals for H^B6^ and H^D6^ which shift to higher frequency on going from [Ir(Ph_2_ppy)_2_(**1**)]^+^ to [Ir(Ph_2_ppy)_2_(**2**)]^+^ (Fig. 6a and b). The chemical shifts for H^B6^ and H^D6^ in [Ir(Ph_2_ppy)_2_(**2**)]^+^ are similar to those in [Ir(Phppy)_2_(**2**)]^+^, indicating that similar effects are operative in both complexes. The effect of cooling a CD_2_Cl_2_ solution of [Ir(Ph_2_ppy)_2_(**2**)][PF_6_] is shown in Fig. S7 and S8.†

### Electrochemical behaviour of [Ir(C^N)_2_(N^N)][PF_6_] complexes

**Fig. 7:**
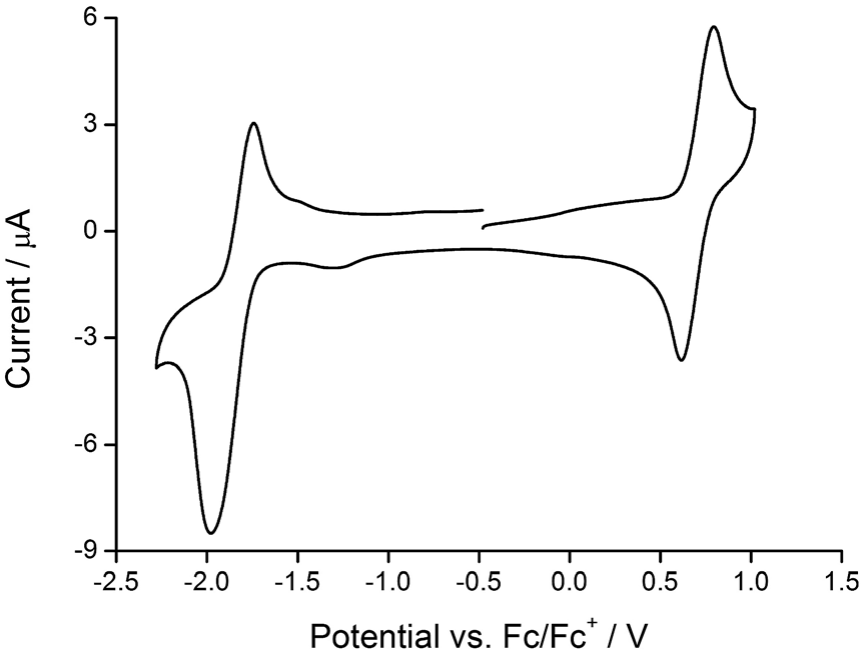
Cyclic voltammogram of [Ir(Ph_2_ppy)_2_(**2**)][PF_6_] (degassed CH_2_Cl_2_ solution) with respect to Fc/Fc^+^; scan rate = 0.1 V s^–1^.

The redox activity of the iridium(iii) complexes was investigated by cyclic voltammetry; data are given in Table 2 and a representative CV is shown in Fig. 7. Each complex exhibits a reversible metal-centred oxidation. The trends in the iridium-centred oxidation potential are consistent with the introduction of electron-releasing phenyl and/or *tert*-butyl groups. In [Ir(ppy)_2_(**1**)][PF_6_], *E*ox1/2 occurs at +0.84 V (*versus* Fc/Fc^+^, in DMF)^22^ and the process occurs at increasingly lower potential on going to [Ir(Phppy)_2_(**1**)]^+^ (+0.79 V) and to [Ir(Ph_2_ppy)_2_(**1**)]^+^ (+0.75 V). This is consistent with a lowering of the HOMO (localized on the C^N ligand and iridium) as sequential electron-releasing phenyl substituents are introduced into the cyclometalating ligand. Substituent effects in the bpy domain also affect the metal oxidation. Table 2 also shows that there is a lowering of *E*ox1/2 on going from [Ir(Phppy)_2_(**1**)]^+^ to [Ir(Phppy)_2_(**2**)]^+^ (Ph introduced to bpy domain), from [Ir(Phppy)_2_(**1**)]^+^ to [Ir(Phppy)_2_(**3**)]^+^ to [Ir(Phppy)_2_(**4**)]^+^ (effect of ^*t*^Bu and of phenyl groups), and similarly from [Ir(Ph_2_ppy)_2_(**1**)]^+^ to [Ir(Ph_2_ppy)_2_(**2**)]^+^, and from [Ir(Ph_2_ppy)_2_(**1**)]^+^ to [Ir(Ph_2_ppy)_2_(**3**)]^+^ to [Ir(Ph_2_ppy)_2_(**4**)]^+^.

**Table 2:** Cyclic voltammetric data with respect to Fc/Fc^+^; CH_2_Cl_2_ solutions with [^*n*^Bu_4_N][PF_6_] supporting electrolyte, and scan rate of 0.1 V s^–1^ (ir = irreversible; qr = quasi-reversible)

Compound	*E* ox 1/2 /V	*E* red 1/2 /V	Δ*E*_1/2_/V
[Ir(Phppy)_2_(**1**)][PF_6_]	+0.79	–1.84^qr^	2.63
[Ir(Phppy)_2_(**2**)][PF_6_]	+0.74	–1.84^qr^	2.58
[Ir(Phppy)_2_(**3**)][PF_6_]	+0.75	–1.88^qr^	2.63
[Ir(Phppy)_2_(**4**)][PF_6_]	+0.72	–1.89^qr^	2.61
[Ir(Ph_2_ppy)_2_(**1**)][PF_6_]	+0.75	–1.82^qr^	2.57
[Ir(Ph_2_ppy)_2_(**2**)][PF_6_]	+0.72	–1.85^qr^	2.57
[Ir(Ph_2_ppy)_2_(**3**)][PF_6_]	+0.71	–1.88^qr^	2.59
[Ir(Ph_2_ppy)_2_(**4**)][PF_6_]	+0.69	–1.91^qr^	2.60

Each complex shows a quasi-reversible reduction (Table 2) assigned to reduction of the bpy ligand (the LUMO is localized on the bpy domain). The value of *E*red1/2 shifts to more negative potential upon introducing ^*t*^Bu substituents, consistent with previous observations.^23^

### Photophysical properties of [Ir(C^N)_2_(N^N)][PF_6_] complexes

**Fig. 8:**
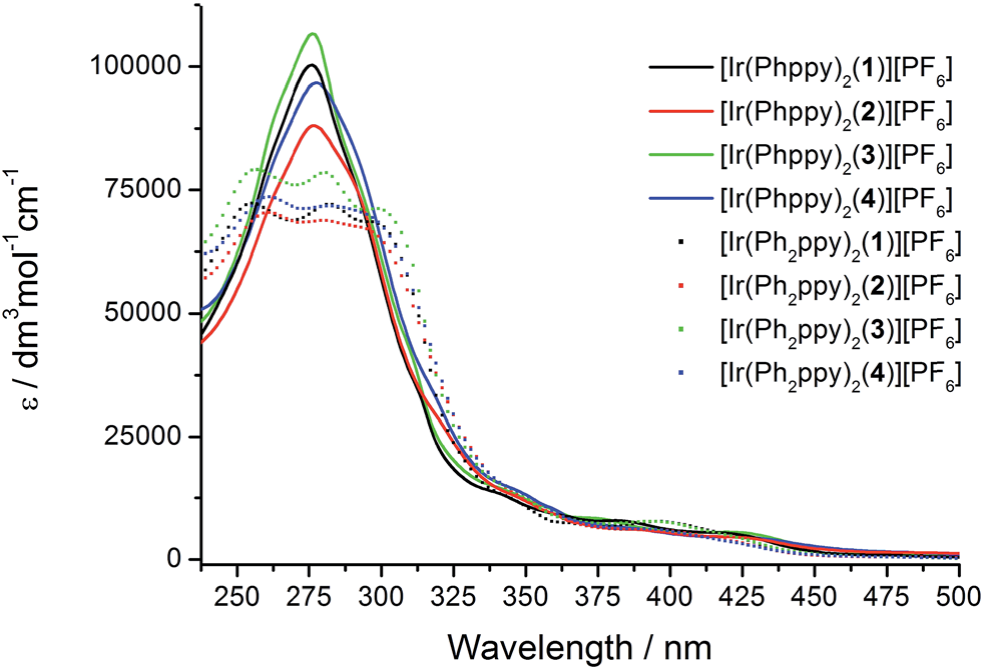
Solution absorption spectra of the [Ir(C^N)_2_(N^N)][PF_6_] complexes (CH_2_Cl_2_, 1 × 10^–5^ mol dm^–3^).

The absorption spectra of CH_2_Cl_2_ solutions of the complexes are shown in Fig. 8. The [Ir(Phppy)_2_(N^N)][PF_6_] family shows an intense, broad absorption with *λ*_max_ in the range 276–278 nm arising from spin-allowed ligand-centred π*←π transitions. For the four [Ir(Ph_2_ppy)_2_(N^N)][PF_6_] complexes, the corresponding bands are broader and exhibit two or three maxima in the approximate range 250–300 nm. The weaker absorptions around 400 and 420 nm are assigned to MLCT transitions. Excitation into the MLCT bands results in broad, unstructured emissions which are, in solution, centred at 600 nm for [Ir(Phppy)_2_(**1**)][PF_6_] and 611 nm for [Ir(Ph_2_ppy)_2_(**1**)][PF_6_] (Table 3). The red-shift in the emission is consistent with destabilization of the HOMO as the electron-releasing phenyl group is introduced into the C^N ligand. An analogous red-shift is observed on going from [Ir(Phppy)_2_(**2**)][PF_6_] to [Ir(Ph_2_ppy)_2_(**2**)][PF_6_], from [Ir(Phppy)_2_(**3**)][PF_6_] to [Ir(Ph_2_ppy)_2_(**3**)][PF_6_], and from [Ir(Phppy)_2_(**4**)][PF_6_] to [Ir(Ph_2_ppy)_2_(**4**)][PF_6_] (Table 3). For both the [Ir(Phppy)_2_(N^N)][PF_6_] and [Ir(Ph_2_ppy)_2_(N^N)][PF_6_] series of complexes, introducing the 6-phenyl substituent into the bpy domain leads to a red-shift in the emission, while introducing the *tert*-butyl groups into the 4- and 4′-positions results in a blue shift (Table 3).

**Table 3:** Emission maxima^*a*^ and quantum yields for [Ir(C^N)_2_(N^N)][PF_6_]

Complex cation	CH_2_Cl_2_ solution		Powder		Thin film^*c*^	
	*λ* max em	PLQY^*b*^/%	*λ* max em	PLQY/%	*λ* max em	PLQY/%
[Ir(Phppy)_2_(**1**)]^+^	600	13	590	30	599	20
[Ir(Phppy)_2_(**2**)]^+^	611	4	596	11	615	11
[Ir(Phppy)_2_(**3**)]^+^	577	35	520	13	592	24
[Ir(Phppy)_2_(**4**)]^+^	590	13	531	13	597	15
[Ir(Ph_2_ppy)_2_(**1**)]^+^	611	8	600	28	614	17
[Ir(Ph_2_ppy)_2_(**2**)]^+^	645	2	570	26	618	7
[Ir(Ph_2_ppy)_2_(**3**)]^+^	588	23	571	56	596	23
[Ir(Ph_2_ppy)_2_(**4**)]^+^	609	4	548	56	602	14

^*a*^Solution: *λ*_exc_ = 420 nm; 400 nm for [Ir(Ph_2_ppy)_2_(**2**)_2_][PF_6_]; solid state: *λ*_exc_ = 400 nm.

^*b*^Argon degassed, 1.00 × 10^–5^ mol dm^–3^.

^*c*^100 nm films of the iridium complex and ionic liquid 1-butyl-3-methyl-imidazolium hexafluoridophosphate ([BMIM][PF_6_]) at a molar ratio of 4 : 1, excitation = 380 nm.

**Fig. 9:**
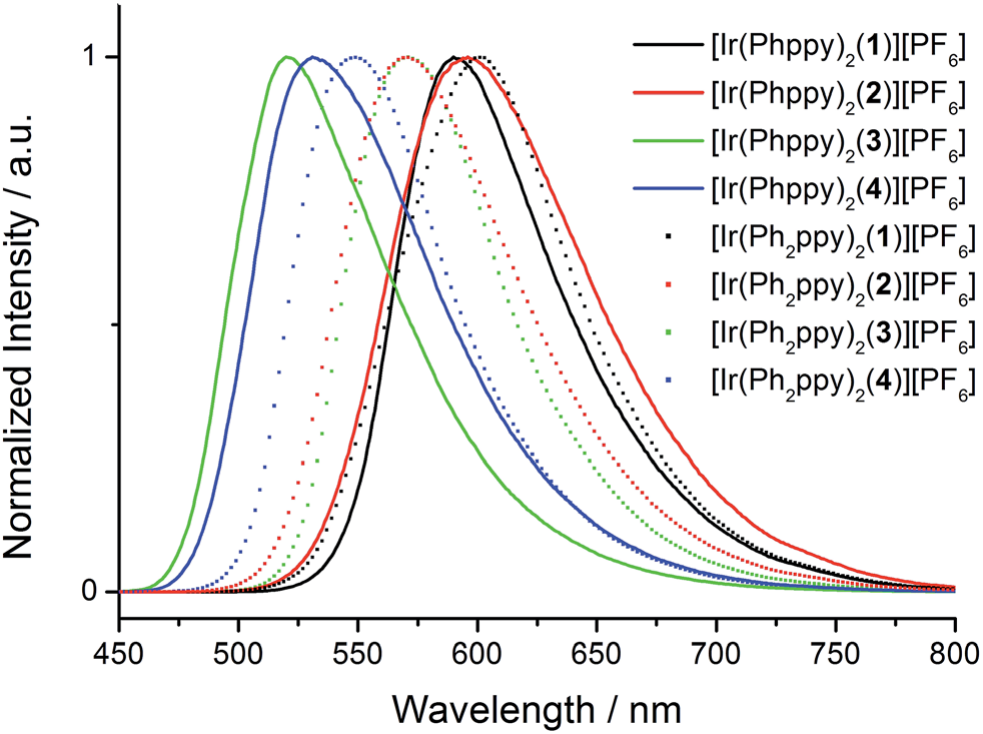
Emission spectra of powdered samples of the [Ir(C^N)_2_(N^N)][PF_6_] complexes (*λ*_exc_ = 400 nm).

**Fig. 10:**
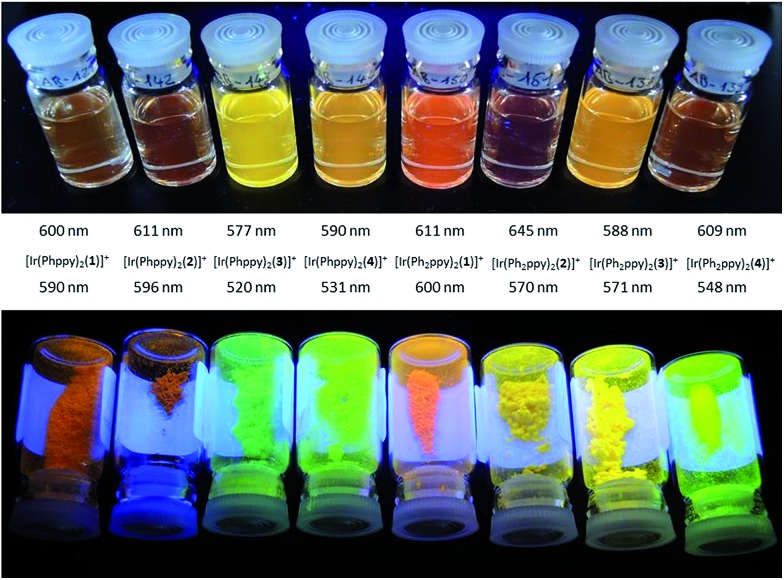
Emission behaviour (*λ*_exc_ = 365 nm) of the [Ir(C^N)_2_(N^N)][PF_6_] complexes in solution (top) and solid state (bottom).

The emission spectra of powdered samples of the complexes were recorded and are presented in Fig. 9. In each case, a blue shift in the emission is observed compared to the solution spectrum (Table 3 and Fig. 10). As in solution, the emission undergoes a red-shift on introducing the additional phenyl group in the cyclometalating ligand in [Ir(Phppy)_2_(**1**)]^+^, [Ir(Phppy)_2_(**3**)]^+^ or [Ir(Phppy)_2_(**4**)]^+^, although a blue-shift is observed on going from [Ir(Phppy)_2_(**2**)]^+^ to [Ir(Ph_2_ppy)_2_(**2**)]^+^ which may be a consequence of packing effects in the solid state in the sterically crowded [Ir(Ph_2_ppy)_2_(**2**)]^+^. Both solution and solid-state emission data confirm that the introduction of the *tert*-butyl groups into the N^N ligand results in significant blue-shifts in *λ*maxem.

The photoluminescence (PL) data for the complexes in the device configuration (thin film), but without electrodes and PEDOT:PSS, are given in Table 3. The similarity between the emission maxima for a given complex in thin film and solution is in contrast to the significant blue shifts observed for most complexes on going from solution to the solid state. This suggests that packing effects may be dominant in determining the latter, since the complex is present in the films only in low concentration. The emission maxima for films of the [Ir(Ph_2_ppy)_2_(N^N)]^+^ complexes are slightly red-shifted compared to those of films [Ir(Phppy)_2_(N^N)]^+^. In each set of complexes, the presence of *tert*-butyl substituents causes a blue-shift in the emission maxima.

The photoluminescence quantum yields (PLQY) are generally enhanced on going from solution to the solid state (Table 3). The four complexes in which C^N = Ph_2_ppy exhibit the highest PLQY values. Lifetimes of the emissions are given in Table 4. For each complex, the luminescence decay was fitted using a biexponential function. Going from solution to the solid state generally results in an increase in the emission lifetime. This is especially noteworthy for the most sterically crowded cations [Ir(Ph_2_ppy)_2_(**2**)]^+^ and [Ir(Ph_2_ppy)_2_(**4**)]^+^ which exhibit values of *τ*_ave_ of 617 and 1148 ns in the solid state compared to 36 and 88 ns, respectively, in argon-degassed solution.

**Table 4:** Photoluminescence lifetimes for [Ir(C^N)_2_(N^N)][PF_6_] (excitation = 280 nm)

Complex cation	CH_2_Cl_2_ solution^*a*^			Powder		
	*τ*_ave_/ns^*b*^	*τ*_1_/ns (*A*_1_)	*τ*_2_/ns (*A*_2_)	*τ*_ave_/ns^*b*^	*τ*_1_/ns (*A*_1_)	*τ*_2_/ns (*A*_2_)
[Ir(Phppy)_2_(**1**)]^+^	260	257 (54 164)	658 (346)	464	457 (80 530)	1011 (1068)
[Ir(Phppy)_2_(**2**)]^+^	101	99 (40 267)	208 (657)	309	305 (59 393)	1207 (252)
[Ir(Phppy)_2_(**3**)]^+^	522	472 (35 392)	573 (34 561)	364	331 (25 592)	1109 (1144)
[Ir(Phppy)_2_(**4**)]^+^	266	265 (46 403)	740 (130)	383	368 (28 153)	1118 (570)
[Ir(Ph_2_ppy)_2_(**1**)]^+^	166	164 (44 021)	314 (711)	591	584 (1329)	1329 (514)
[Ir(Ph_2_ppy)_2_(**2**)]^+^	36	28 (6400)	39 (18 317)	617	611 (44 188)	2204 (162)
[Ir(Ph_2_ppy)_2_(**3**)]^+^	322	368 (35 110)	189 (12 273)	806	791 (71 945)	3088 (480)
[Ir(Ph_2_ppy)_2_(**4**)]^+^	88	87 (41 551)	373 (80)	1148	1105 (61 273)	1761 (4344)

^*a*^Argon degassed, 1.00 × 10^–5^ mol dm^–3^.

^*b*^Biexponential fit using the equation *τ*_ave_ = ∑*A*_*i*_*τ*_*i*_/∑*A*_*i*_ where *A*_*i*_ is the pre-exponential factor for the lifetime.

### LEC performances and electroluminescence

**Fig. 11:**
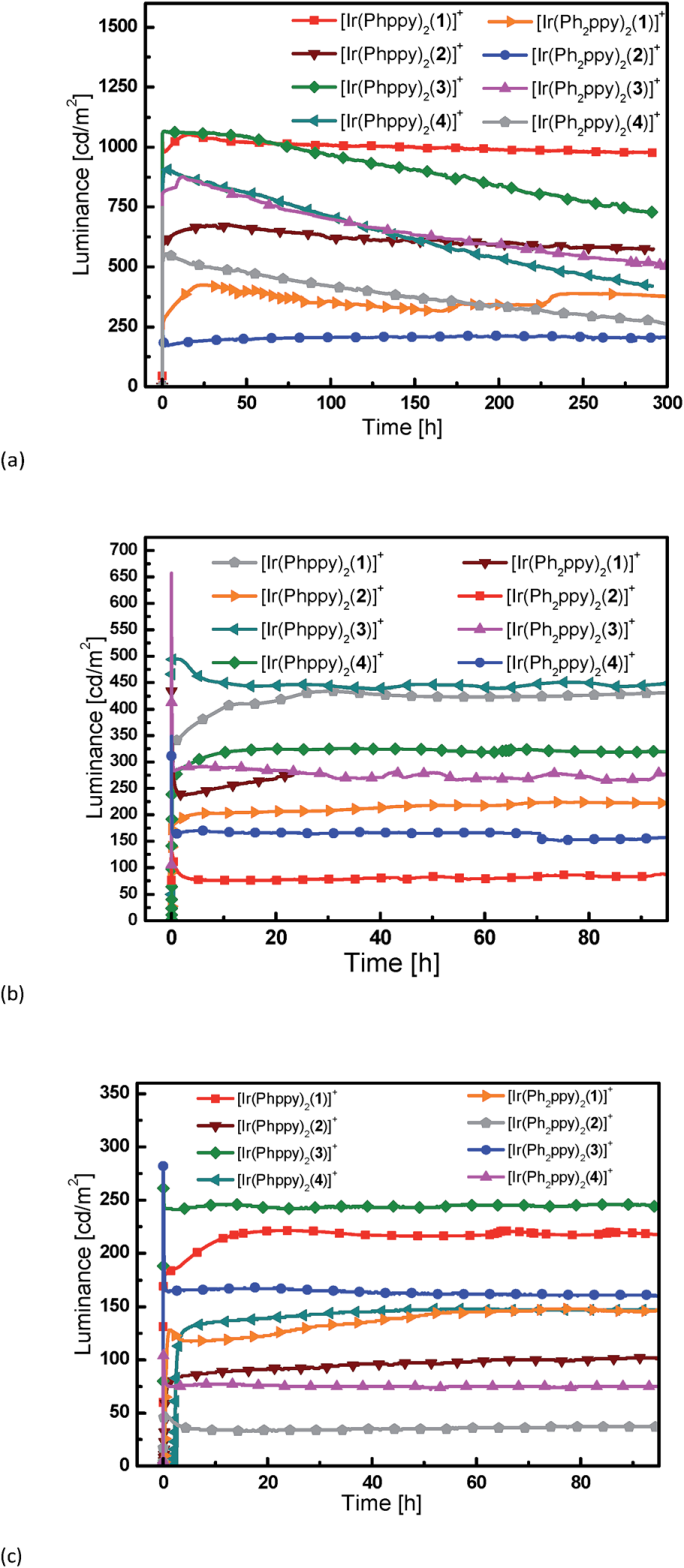
Luminance *vs.* time for the LECs driven using pulsed current driving (a) at 300 A m^–2^, (b) at 100 A m^–2^ and (c) at 50 A m^–2^, all at a frequency of 1 kHz and duty cycles of 50%.

Simple LECs were prepared using all complexes, the devices were prepared on ITO-coated glass plates and consisted of a PEDOT:PSS hole injection/planarization layer (60 nm), the light-emitting layer, consisting of the iridium complex and 1-butyl-3-methyl-imidazolium hexafluoridophosphate ([BMIM][PF_6_]) at a molar ratio of 4 : 1, and an aluminum top electrode. Fig. 11 and S9† show plots of luminance and operating voltage *vs.* time for the devices containing the eight [Ir(C^N)_2_(N^N)][PF_6_] complexes when a pulsed current density of 300 A m^–2^ is applied. Device parameters are given in Table 5. The turn-on time for the devices varies considerably. It is relatively short (29 seconds) for the device containing [Ir(Ph_2_ppy)_2_(**4**)]^+^ increasing to over an hour for LECs employing [Ir(Phppy)_2_(**4**)]^+^ and [Ir(Ph_2_ppy)_2_(**1**)]^+^. The voltage behaviour is typical for LEC devices, starting at a high value due to an initial high injection barrier and rapidly dropping as a result of the electric double-layer formation that reduces the injection barriers^24^ (Fig. S9†).

**Table 5:** LEC device parameters obtained by applying a pulsed current density of 300 A m^–2^, at a frequency of 1 kHz and duty cycles of 50%. *t*_on_ = time to reach maximum luminescence; *t*_1/2_ = time to reach half of the maximum luminance

Complex cation	Luminance_max_/cd m^–2^	Efficacy_max_/cd A^–1^	*t*_on_/h	*t*_1/2_/h
[Ir(Phppy)_2_(**1**)]^+^	1024	3.5	0.14	2800
[Ir(Phppy)_2_(**2**)]^+^	676	2.2	0.42	1204
[Ir(Phppy)_2_(**3**)]^+^	1090	3.5	0.03	437
[Ir(Phppy)_2_(**4**)]^+^	910	2.9	1.11	260
[Ir(Ph_2_ppy)_2_(**1**)]^+^	425	1.4	1.21	360
[Ir(Ph_2_ppy)_2_(**2**)]^+^	261	0.7	0.05	>2800
[Ir(Ph_2_ppy)_2_(**3**)]^+^	1048	2.9	0.07	282
[Ir(Ph_2_ppy)_2_(**4**)]^+^	748	1.8	0.01	147

Most LECs reported in the literature have been driven using a constant voltage mode. However, this leads to an increase of the width of the doped zone over time. As doped materials are efficient exciton quenchers, this leads to (partially reversible) reduction in the luminance.^7,18^ To avoid this decrease in performance, we have driven the devices using a pulsed current mode, with a frequency of 1 kHz and a duty cycle of 50%.^25,26^ Using pulsed current driving, iridium iTMC-based LECs are usually operated at an average current density of 50 or 100 A m^–2^. The luminance and voltage *versus* time curves for the different devices (tested at 50 and 100 A m^–2^) are depicted in Fig. 11 and S9,† respectively. For a number of devices, the luminance does not appear to decay over time. This is obviously a good property, yet does not allow an analysis of the relationship between iridium complex composition and the device performance. Therefore, to distinguish between the different LECs, all devices were also driven using a much higher current density, of 300 A m^–2^, which permits acceleration of the degradation of the device due to the higher stress that the materials are subjected to. The luminance increases with higher current density although not linearly. This is due to a reduction in device efficiency as a result of charge induced carrier quenching.^27,28^

The devices containing the [Ir(Ph_2_ppy)_2_(N^N)]^+^ iTMCs have a slightly lower luminance than those based on [Ir(Phppy)_2_(N^N)]^+^. Under these pulsed current conditions, the efficiency scales directly to the luminance, and it follows that the efficiencies are also lower for the [Ir(Ph_2_ppy)_2_(N^N)]^+^ complexes. The effect of introducing the *tert*-butyl groups in the N^N domain does not lead to an increase in luminance or in the efficiency of the LECs as might be expected by comparison with previous results.^11^ This is probably related to the fact that the [Phppy]^–^ and [Ph_2_ppy]^–^ ligands are sterically demanding which results in reduced close packing in the film, thereby enhancing the radiative decay pathways. The efficiency of the LEC devices containing N^N ligands **2** or **4** with the 6-phenyl group is lower than those in which N^N = bpy; this is consistent with previous results.^11,29^ This is directly related to a lower PLQY of the complexes that exhibit the π–π stacking.

The trend in the lifetimes of the devices is both important and interesting. In general, the devices based on iTMCs that contain the phenyl group on the bpy ligand, and hence show intra-cation π–π stacking, show a faster decay of luminance than the devices using the iTMCs without the π–π stacking ability. However, this trend is not observed for the device containing [Ir(Ph_2_ppy)_2_(**2**)]^+^ which, although exhibiting a rather low luminance of 200 cd m^–2^, stays constant over a period of 350 hours (Fig. 11a).

The series of iTMCs evaluated in this study all exhibit exceptional stabilities in LECs. The best performances are observed for devices containing [Ir(Phppy)_2_(**1**)][PF_6_], with a maximum efficiency of 3.5 cd A^–1^ and luminance of 1024 cd m^–2^ (at an average current density of 300 A m^–2^) and an extrapolated lifetime in excess of 2800 hours (time to reach 50% of the maximum luminance). In [Ir(Phppy)_2_(**1**)][PF_6_], the phenyl substituents on the C^N ligand reside on the periphery of the complex (Fig. 1) and are not involved in inter-ligand π-stacking within the iridium(iii) coordination sphere. Although incorporation of an intra-cation π-stacking domain may be advantageous,^7,9,10^ this is not necessarily a general design principle^8,19^ and in the current study, the presence of intra-cation π-stacking does not improve the stability of the light emitting device.

The electroluminescence spectra (Fig. S10†) are slightly blue shifted with respect to the photoluminescence maxima as reported in Table 3.

## Conclusions

We have designed a series of cyclometalated iridium(iii) complexes [Ir(C^N)_2_(N^N)]^+^ in order to examine the effects of having multiple π-stacking domains within the coordination sphere of the iridium(iii) centre. The complexes have been synthesized *via* solvento precursors, thus avoiding the use of chlorido-dimer intermediates. Single crystal structure determinations and variable temperature solution ^1^H NMR spectroscopic data confirm that the pendant phenyl domains engage in multiple face-to-face π-interactions. The [Ir(Phppy)_2_(N^N)]^+^ and [Ir(Ph_2_ppy)_2_(N^N)]^+^ iTMCs all show excellent luminescent properties, in particular when employed in thin solid films. LECs using these complexes exhibit a very stable luminance output over time even when driven at elevated current densities. The most stable LEC had an extrapolated lifetime in excess of 2500 hours at a starting luminance above 1000 cd m^–2^ achieved under accelerated testing conditions. These remarkable lifetimes were obtained for devices using complexes both with and without the ability to form intra-molecular face-to-face π-stacking.

## Supplementary Material

Supplementary information

Crystal structure data
