# Supplementary material for: Exceptionally long-lived light-emitting electrochemical cells: multiple intra-cation π-stacking interactions in [Ir(C^N)2(N^N)][PF6] emitters
Source: Chem Sci. 2015 Mar 6;6(5):2843–52. doi: 10.1039/c4sc03942d (PMC5654370; doi:10.1039/c4sc03942d)
Supplement: Supplementary file 1 [file SC-006-C4SC03942D-s001.pdf]

Electronic Supplementary Material (ESI) for Chemical Science.
This journal is © The Royal Society of Chemistry 2015

ESI to accompany

Exceptionally long-lived light-emitting electrochemical cells: multiple intra-cation π-stacking interactions in $[Ir(C^\wedge N)_2(N^\wedge N)][PF_6]$ emitters

Andreas M. Bünzli,[a] Edwin C. Constable,[a] Catherine E. Housecroft,[a]* Alessandro Prescimone,[a] Jennifer A. Zampese,[a] Giulia Longo,[b] Lidón Gil-Escrig,[b] Antonio Pertegás,[b] Enrique Ortí,[b] Henk J. Bolink*[b,c]

Experimental

^{1}H and ^{13}C NMR spectra were recorded on a Bruker Avance III-500 or III-600 NMR spectrometer (chemical shifts with respect to δ(TMS) = 0 ppm). Solution electronic absorption and emission spectra were recorded on an Agilent 8453 spectrophotometer and Shimadzu 5301PC spectrofluorophotometer, respectively; a Perkin Elmer Spectrum Two UATR instrument was used to record FT-IR spectra of solid samples. Electrospray ionization (ESI) mass spectra were measured using a Bruker esquire 3000^{plus} mass spectrometer. Solution and solid-state quantum yields were measured using a Hamamatsu absolute PL quantum yield spectrometer C11347 Quantaurus_QY. Lifetimes and emission spectra of powdered samples were measured using a Hamamatsu Compact Fluorescence lifetime Spectrometer C11367 Quantaurus-Tau.

Electrochemical measurements were performed using a CH Instruments 900B potentiostat using a glassy carbon working electrode, platinum-wire auxiliary electrode, and silver-wire pseudo-reference electrode. The redox potentials were determined by both cyclic voltammetry (CV) and square wave voltammetry. HPLC grade, argon degassed CH_2Cl_2 solutions ($\approx 10^{-4}$ mol dm^{-3}) of samples were used in the presence of 0.1 M [^{n}Bu$_4$N][PF$_6$] as supporting electrolyte; a scan rate of 0.1 V s^{-1} and ferrocene (Fc$^+$/Fc) was used as an internal standard.

The compounds HPhppy[1] and HPh$_2$ppy[1,2] were made according to literature methods. Atom labelling for NMR assignments is shown in Scheme S1.

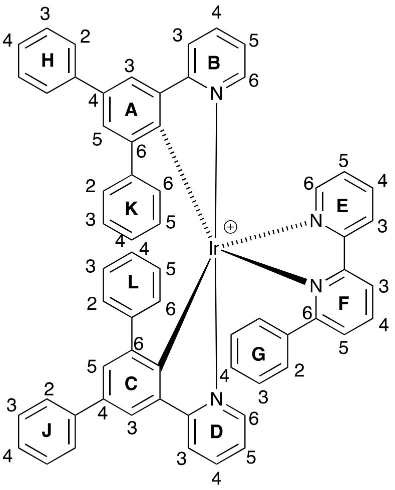

Scheme S1. Ring and atom labelling in $[Ir(Ph_2ppy)_2(\mathbf{2})]^+$. Analogous ring labelling is used in all complex cations.

$[Ir_2(Phppy)_4Cl_2]$

A mixture of H_2O (8 mL) and 2-ethoxyethanol (25 mL) containing HPhppy (0.60 g, 2.59 mmol) and $IrCl_3{\cdot}xH_2O$ (6.38, 1.13 mmol) was heated at 115 °C for 20 h. The reaction mixture was allowed to reach room temperature, and then the yellow precipitate was removed by filtration, washed with H_2O and n-hexane and then redissolved in acetone to be purified by column chromatography (Fluka silica gel 60, 0.040–0.063 mm; CH_2Cl_2:iPrOH 100:1). $[Ir_2(Phppy)_4Cl_2]$ was isolated as a yellow solid (0.362 g, 0.263 mmol, 47%). ^1H NMR (500 MHz, acetone-d_6) δ/ ppm 9.34 (ddd, J = 5.8, 1.5, 0.7 Hz, 4H, H^{B6}), 8.41 (d, J = 8.0 Hz, 4H, H^{B3}), 8.06 (ddd, J = 8.1, 7.6, 1.6 Hz, 4H, H^{B4}), 7.97 (d, J = 2.0 Hz, $4H^{A3}$), 7.55 (m, 8H, H^{H2}), 7.33 (m, 8H, H^{H3}), 7.22 (m, 4H, H^{H4}), 7.11 (ddd, J = 7.3, 5.7, 1.4 Hz, H^{B5}), 6.86 (dd, J = 8.1, 2.0 Hz, 4H, H^{A5}), 6.03 (d, J = 8.1 Hz, 4H, H^{A6}). ^{13}C NMR (126 MHz, acetone-d_6) δ/ppm 168.9 (C^{B2}), 152.3 (C^{B6}), 146.0 (C^{A2}), 145.8 (C^{A1}), 142.3 (C^{H1}), 138.3 (C^{B4}), 135.1 (C^{A4}), 131.8 (C^{A6}), 129.5 (C^{H3}), 128.3 (C^{A5}), 127.2 (C^{H4}), 127.1 (C^{H2}), 124.1 (C^{B5}), 123.0 (C^{A3}), 120.2 (C^{B3}). The complex was used in the next step without further characterization.

$[Ir_2(Ph_2ppy)_4Cl_2]$

A mixture of H_2O (5 mL) and 2-ethoxyethanol (15 mL) containing HPh_2ppy (0.50 g, 1.63 mmol) and $IrCl_3{\cdot}xH_2O$ (0.40 g, 0.707 mmol) was heated at 110 °C for 20 h under inert atmosphere. The reaction mixture was allowed to reach room temperature, and then the yellow precipitate was collected by filtration, washed with H_2O and Et_2O and then redissolved in CH_2Cl_2. It was purified by column chromatography (Fluka silica gel 60, CH_2Cl_2 changing to CH_2Cl_2 : MeOH 100 : 3). $[Ir_2(Ph_2ppy)_4Cl_2]$ was isolated as a yellow solid (0.342 g, 0.203 mmol, 58 %). ^1H NMR (500 MHz, acetone-d_6) δ/ ppm 8.02 (dd, J = 5.8, 1.1 Hz, 4H, H^{B6}), 7.66 (d, J = 2.0 Hz, 4H, H^{A3}), 7.60 (m, 8H, H^{H2}), 7.37 (m, 8H, H^{H3}), 7.34 (d, J = 8.1 Hz, 4H, H^{B3}),

7.26 (m, 4H, H^{H4}), 7.10 (ddd, J = 8.2, 7.4, 1.5 Hz, 4H, H^{B4}), 7.05 (m, 4H, $H^{K2/K6}$), 6.89 (d, J = 2.1 Hz, 4H, H^{A5}), 6.84 (broadened t, $J \approx$ 6.8 Hz, 4H, $H^{K3/K5}$), 6.67 (tt, J = 7.3, 1.3 Hz, 4H, H^{K4}), 6.27 – 6.15 (m, 8H, $H^{K2/K6+K3/K5}$), 5.86 (ddd, J = 7.2, 5.8, 1.3 Hz, 4H, H^{B5}). ^{13}C NMR (126 MHz, acetone-d$_6$) δ / ppm 167.2 (C^{B2}), 151.3 (C^{B6}), 148.7 (C^{A2}), 148.4 (C^{A6}), 145.0 (C^{K1}), 142.7 (C^{A1}), 141.4 (C^{H1}), 135.6 (C^{B4}), 134.7 (C^{A4}), 130.7 (C^{A5}), 128.7 (C^{H3}), 128.4 ($C^{K2/K6}$), 126.8 ($C^{K3/K5}$), 126.6 (C^{H2}), 125.2 (C^{H4}), 121.9 (C^{B5}), 120.9 (C^{A3}), 118.6 (C^{B3}). The complex was used in the next step without further characterization.

[Ir(Phppy)$_2$(MeOH)$_2$][PF$_6$]

A suspension of [Ir$_2$(Phppy)$_4$Cl$_2$] (1.15 g, 0.838 mmol) and AgPF$_6$ (0.464 g, 1.84 mmol) in MeOH (70 mL) was stirred at room temperature for 2 h. The precipitated AgCl was removed by filtration over Celite. Evaporation of the yellow filtrate under reduced pressure gave [Ir(Phppy)$_2$(MeOH)$_2$][PF$_6$] as a yellow solid (1.43 g, 1.66 mmol, 99%). ^{1}H NMR (500 MHz, CD$_3$OD) δ / ppm 8.92 (d, J = 5.0 Hz, 2H, H^{B6}), 8.28 (d, J = 8.1 Hz, 2H, H^{B3}), 8.07 (m, 2H, H^{B4}), 7.93 (d, 2H, H^{A3}), 7.59–7.46 (m, 6H, H^{B5+H2}), 7.35 (t, J = 7.7 Hz, 4H, H^{H3}), 7.24 (t, J = 7.4 Hz, 2H, H^{H4}), 6.96 (dd, J = 8.0, 1.9 Hz, 2H, H^{A5}), 6.18 (d, J = 7.9 Hz, 2H, H^{A6}), 3.35 (s, 6H, H^{Me}). ^{13}C NMR (126 MHz, CD$_3$OD) δ / ppm 169.5 (C^{B2}), 150.2 (C^{B6}), 146.7 (C^{A2}), 142.4 (C^{H1}), 140.4 (C^{B4}), 138.2 (C^{A1}), 137.1 (C^{A4}), 134.5 (C^{A6}), 129.8 (C^{H3}), 129.4 (C^{A5}), 127.8 (C^{H4}), 127.6 (C^{H2}), 124.2 (C^{B5}), 123.8 (C^{A3}), 120.8 (C^{B3}), 49.9 (C^{Me}). ^{31}P NMR (202 MHz, CD$_3$OD) δ / ppm –144.6 (septet, J_{PF} = 707 Hz). The complex was used immediately in the next step without further characterization.

[Ir(Ph$_2$ppy)$_2$(MeOH)$_2$][PF$_6$]

The method was as for [Ir(Phppy)$_2$(MeOH)$_2$][PF$_6$] starting with [Ir$_2$(Ph$_2$ppy)$_4$Cl$_2$] (298 mg, 0.177 mmol) and AgPF$_6$ (98.2 mg, 0.388 mmol). [Ir(Ph$_2$ppy)$_2$(MeOH)$_2$][PF$_6$] was isolated as a yellow-green solid (358 mg, 0.353 mmol, 99%). ^{1}H NMR (500 MHz, CD$_3$OD) δ / ppm 8.23 (ddd, J = 5.9, 1.4, 0.6 Hz, 2H, H^{B6}), 7.87 (d, J = 2.1 Hz, 2H, H^{A3}), 7.81 (d, J = 8.2 Hz, 2H, H^{B3}), 7.70–7.64 (m, 6H, H^{H2+B4}), 7.43 (m, 4H, H^{H3}), 7.31 (tt, J = 7.0, 1.2 Hz, 2H, H^{H4}), 7.07 (ddd, J = 7.3, 5.8, 1.3 Hz, 2H, H^{B5}), 6.98 (broadened d, $J \approx$ 6.8 Hz, 2H, $H^{K2/K6}$), 6.94 (d, J = 2.1 Hz, 2H, H^{A5}), 6.91–6.82 (overlapping m, 4H, H^{K3+K5}), 6.55 (broadened t, $J \approx$ 6.8 Hz, 2H, H^{K4}), 6.28 (broadened d, $J \approx$ 7.5 Hz, 2H, $H^{K2/K6}$), 3.35 (s, 6H, H^{Me}). ^{13}C NMR (126 MHz, CD$_3$OD) δ / ppm 168.6 (C^{B2}), 153.4 (C^{A6}), 151.0 (C^{B6}), 150.3 (C^{A2}), 146.3 (C^{K1}), 141.9 (C^{H1}), 139.4 (C^{B4}), 137.0 (C^{A4}), 132.0 (C^{A1}), 131.0 (C^{A5}), 130.0 ($C^{K2/K6}$), 130.0 (C^{H3}), 129.6 ($C^{K2/K6}$), 128.2 ($C^{K3/K5}$), 128.1 (C^{H4}), 127.7 (C^{K4}), 127.5 (C^{H2}), 127.1 ($C^{K3/K5}$), 122.8 (C^{B5}), 122.7 (C^{A3}), 120.8 (C^{B3}), 49.9 (C^{Me}). ^{31}P NMR (202 MHz, CD$_3$OD) δ / ppm –144.6 (septet, J_{PF} = 707 Hz). The complex was used immediately in the next step.

[Ir(Phppy)$_2$(1)][PF$_6$]

A suspension of [Ir(Phppy)$_2$(MeOH)$_2$][PF$_6$] (0.150 g, 0.174 mmol) and **1** (27.5 mg, 0.176 mmol) in MeOH (15 mL) was sonicated until all the solid had dissolved. An excess of solid NH$_4$PF$_6$ (10 eq) was added to the solution; this was then stirred for 1 h at room temperature.

The yellow precipitate was separated by filtration, washed with MeOH and Et_2O, and then purified by column chromatography (Fluka silica gel 60, CH_2Cl_2 changing to CH_2Cl_2 : MeOH 100 : 0.5). Orange, crystalline [Ir(Phppy)$_2$(**1**)][PF$_6$] was obtained from a CH_2Cl_2 solution of the product overlaid with EtOH : n-hexane 2 : 1 (154 mg 0.161 mmol, 93%). ^{1}H NMR (500 MHz, CD_2Cl_2) δ / ppm 8.54 (d, J = 8.1 Hz, 2H, H^{E3}), 8.19–8.11 (m, 4H, H^{E4+E6}), 8.09 (d, J = 8.3 Hz, 2H, H^{B3}), 7.98 (d, J = 2.0 Hz, 2H, H^{A3}), 7.85 (ddd, J = 8.4, 7.6, 1.5 Hz, 2H, H^{B4}), 7.61 (m, 4H, H^{H2}), 7.57 (dd, J = 5.5, 0.8 Hz, 2H, H^{B6}), 7.51 (ddd, J = 7.5, 5.4, 0.9 Hz, 2H, H^{E5}), 7.43 (m, 4H, H^{H3}), 7.33 (m, 2H, H^{H4}), 7.23 (dd, J = 7.9, 1.9 Hz, 2H, H^{A5}), 7.06 (ddd, J = 7.3, 5.8, 1.4 Hz, 2H, H^{B5}), 6.45 (d, J = 7.9 Hz, 2H, H^{A6}). ^{13}C NMR (126 MHz, CD_2Cl_2) δ / ppm 168.1 (C^{B2}), 156.2 (C^{E2}), 151.4 (C^{E6}), 149.6 (C^{A1}), 149.2 (C^{B6}), 144.9 (C^{A2}), 141.6 (C^{H1}), 140.1 (C^{E4}), 138.9 (C^{B4}), 136.5 (C^{A4}), 132.7 (C^{A6}), 130.2 (C^{A5}), 129.4 (C^{H3}), 129.0 (C^{E5}), 127.5 (C^{H4}), 127.1 (C^{H2}), 125.2 (C^{E3}), 124.2 (C^{B5}), 124.0 (C^{A3}), 120.6 (C^{B3}). IR (solid, $\tilde{\nu}$ / cm^{-1}) 3028 (w), 1600 (w), 1563 (w), 1534 (w), 1479 (m), 1461 (w), 1447 (w), 1428 (m), 1313 (w), 1262 (w), 1165 (w), 1073 (w), 1027 (w), 878 (w), 837 (s), 826 (s), 773 (s), 760 (s), 744 (s), 734 (m), 720 (m), 705 (m), 693 (m), 637 (m), 608 (w), 556 (s), 518 (w), 489 (w), 475 (w). UV-Vis (CH_2Cl_2, 1.00 × 10^{-5} mol dm^{-3}) λ / nm (ε / dm^3 mol^{-1} cm^{-1}) 276 (100 000), 295sh (69 000), 315sh (32 000), 345 (13 000), 380 (8 000), 420 (5 000). Emission (CH_2Cl_2, 1.00 × 10^{-5} mol dm^{-3}, λ_{exc} = 420 nm) λ_{em} = 600 sh, 639 nm. ESI-MS m/z 809.6 [M–PF$_6$]$^+$ (base peak, calc. 809.0). Found C 54.71, H 3.57, N 5.93; $C_{44}H_{32}F_6IrN_4P{\cdot}0.5\ H_2O$ requires C 54.88, H 3.45, N 5.82%.

[Ir(Phppy)$_2$(2)][PF$_6$]

The method was as for [Ir(Phppy)$_2$(**1**)][PF$_6$] starting with [Ir(Phppy)$_2$(MeOH)$_2$][PF$_6$] (0.150 g, 0.174 mmol) and **2** (40.8 mg, 0.176 mmol). [Ir(Phppy)$_2$(**2**)][PF$_6$] was isolated as an orange solid (105 mg, 0.102 mmol, 59%). ^{1}H NMR (500 MHz, CD_2Cl_2) δ / ppm 8.56 (dd, J = 8.1, 1.1 Hz, 1H, H^{F3}), 8.53 (d, J = 8.2 Hz, 1H, H^{E3}), 8.24 (t, J = 7.9 Hz, 1H, H^{F4}), 8.14 (td, J = 8.0, 1.5 Hz, 1H, H^{E4}), 8.00 (dd, J = 5.5, 1.1 Hz, 1H, H^{E6}), 7.96 (m, 2H, H^{B3+D3}), 7.92 (m, 1H, H^{B4}), 7.82 (m, 1H, H^{D4}), 7.78 (d, J = 2.0 Hz, 1H, H^{A3}), 7.75 (m, 1H, H^{B6}), 7.55 (m, 2H, H^{H2}), 7.50 (m, 3H, H^{J2+F5}), 7.46 (m, 1H, H^{D6}), 7.45–7.36 (m, 6H, $H^{C3+E5+H3+J3}$), 7.33–7.25 (m, 2H, H^{H4+J4}), 7.17–7.07 (m, 3H, $H^{A5+B5+D5}$), 6.97 (tt, J = 7.5, 1.2 Hz, 1H, H^{G4}), 6.76 (broadened t, 2H, H^{G3}), 6.69 (dd, J = 7.9, 1.9 Hz, 1H, H^{C5}), 6.57 (br, H^{G2}), 6.10 (d, J = 8.0 Hz, 1H, H^{A6}), 5.73 (d, J = 7.9 Hz, 1H, H^{C6}). ^{13}C NMR (126 MHz, CD_2Cl_2) δ / ppm 169.2 (C^{B2}), 167.5 (C^{D2}), 166.4 (C^{F6}), 157.5 (C^{E2}), 157.3 (C^{F2}), 150.9 (C^{E6}), 150.6 (C^{C1}), 149.5 (C^{B6}), 149.5 (C^{D6}), 146.1 (C^{A1}), 144.2 (C^{A2}), 144.0 (C^{C2}), 142.2 (C^{J1}), 141.2 (C^{H1}), 140.3 (C^{F4}), 139.9 (C^{E4}), 138.9 (C^{D4}), 138.8 (C^{B4}), 138.5 (C^{G1}), 136.5 (C^{A4}), 134.3 (C^{C4}), 132.5 (C^{C6}), 131.3 (C^{A6}), 130.7 (C^{F5}), 130.2 (C^{A5}), 129.4 ($C^{H3/J3}$), 129.3 ($C^{H3/J3}$), 129.2 (C^{C5}), 129.0 (C^{G4}), 128.5 (C^{G3}), 128.4 (C^{E5}), 128.1 (C^{G2}), 127.6 (C^{H4}), 127.1 (C^{J4}), 127.0 (C^{H2}), 126.9 (C^{J2}), 125.6 (C^{E3}), 124.3 (C^{D5}), 124.2 (C^{F3}), 123.8 (C^{C3}), 123.7 (C^{A3}), 123.3 (C^{B5}), 120.7 (C^{B3}), 120.5 (C^{D3}). IR (solid, $\tilde{\nu}$ / cm^{-1}) 3027 (w), 1599 (m), 1562 (w), 1535 (w), 1478 (m), 1449 (m), 1428 (m), 1326 (w), 1296 (w), 1253 (w), 1224 (w), 1165 (w), 1113 (w), 1072 (w), 1031 (w), 877 (w), 835 (s), 781 (m), 760 (s), 718 (m), 696 (s), 639 (m), 624 (w), 608 (w), 556 (s), 487 (w). UV-Vis (CH_2Cl_2, 1.00 × 10^{-5} mol dm^{-3}) λ / nm (ε / dm^3 mol^{-1} cm^{-1}) 277 (88 000), 295 sh, (69 000), 315 sh (33 000), 345 (13 000), 380 (6 000),

420 (5 000). Emission (CH_2Cl_2, 1.00 × 10^{-5} mol dm^{-3}, λ_{exc} = 420 nm) λ_{em} = 611, 639 sh nm. ESI-MS *m/z* 885.7 $[M–PF_6]^+$ (base peak, calc. 885.1). Found C 58.00, H 3.90, N 5.37; $C_{50}H_{36}F_6IrN_4P$ requires C 58.30, H 3.52, N 5.44%.

$[Ir(Phppy)_2(3)][PF_6]$

The method was as for $[Ir(Phppy)_2(\mathbf{1})][PF_6]$ starting with $[Ir(Phppy)_2(MeOH)_2][PF_6]$ (0.150 g, 0.174 mmol) and **3** (47.2 mg, 0.176 mmol). $[Ir(Phppy)_2(\mathbf{3})][PF_6]$ was isolated as a yellow solid (148 mg, 0.139 mmol, 80%). ^1H NMR (500 MHz, CD_2Cl_2) δ / ppm 8.31 (d, *J* = 1.7 Hz, 2H, H^{E3}), 8.09 (d, *J* = 8.0 Hz, 2H, H^{B3}), 8.02 (d, *J* = 5.8 Hz, 2H, H^{E6}), 7.97 (d, *J* = 1.9 Hz, 2H, H^{A3}), 7.86 (ddd, *J* = 8.3, 7.6, 1.5 Hz, 2H, H^{B4}), 7.61 (m, 4H, H^{H2}), 7.58 (m, 2H, H^{B6}), 7.48 (dd, *J* = 5.9, 1.9 Hz, 2H, H^{E5}), 7.44 (m, 4H, H^{H3}), 7.33 (m, 2H, H^{H4}), 7.22 (dd, *J* = 7.9, 2.0 Hz, 2H, H^{A5}), 7.08 (ddd, *J* = 7.3, 5.9, 1.3 Hz, 2H, H^{B5}), 6.44 (d, *J* = 7.9 Hz, 2H, H^{A6}), 1.44 (s, 18H, H^{tBu}). ^{13}C NMR (126 MHz, CD_2Cl_2) δ / ppm 168.2 (C^{B2}), 164.7 (C^{E4}), 156.1 (C^{E2}), 151.0 (C^{E6}), 150.1 (C^{A1}), 149.3 (C^{B6}), 144.9 (C^{A2}), 141.7 (C^{H1}), 138.8 (C^{B4}), 136.3 (C^{A4}), 132.6 (C^{A6}), 130.1 (C^{A5}), 129.4 (C^{H3}), 127.5 (C^{H4}), 127.1 (C^{H2}), 126.3 (C^{E5}), 124.0 (C^{B5}), 123.9 (C^{A3}), 121.5 (C^{E3}), 120.5 (C^{B3}), 36.2 ($C^{quat-tBu}$), 30.6 (C^{tBu}). IR (solid, $\tilde{\nu}$ / cm^{-1}) 2958 (w), 1610 (w), 1563 (w), 1478 (m), 1429 (w), 1415 (w), 1368 (w), 1253 (w), 1224 (w), 1166 (w), 1070 (w), 1030 (w), 914 (w), 895 (w), 877 (w), 832 (s), 824 (s), 784 (m), 761 (s), 741 (m), 720 (w), 698 (m), 639 (w), 607 (m), 556 (s), 483 (w). UV-Vis (CH_2Cl_2, 1.00 × 10^{-5} mol dm^{-3}) λ / nm (*ε* / dm^3 mol^{-1} cm^{-1}) 276 (107 000), 295 sh (73 000), 310 sh (41 000), 345 (14 000), 375 (8 000), 420 (5 000). Emission (CH_2Cl_2, 1.00 × 10^{-5} mol dm^{-3}, λ_{exc} = 420 nm) λ_{em} = 577, 639 sh nm. ESI-MS *m/z* 921.8 $[M–PF_6]^+$ (base peak, calc. 921.2). Found C 58.32, H 4.88, N 5.42; $C_{52}H_{48}F_6IrN_4P$ requires C 58.58, H 4.54, N 5.26%.

$[Ir(Phppy)_2(4)][PF_6]$

The method was as for $[Ir(Phppy)_2(\mathbf{1})][PF_6]$ starting with $[Ir(Phppy)_2(MeOH)_2][PF_6]$ (0.150 g, 0.174 mmol) and **4** (60.6 mg, 0.176 mmol). $[Ir(Phppy)_2(\mathbf{4})][PF_6]$ was isolated as a yellow solid (145 mg, 0.127 mmol, 73%). ^1H NMR (500 MHz, CD_2Cl_2) δ / ppm 8.35 (d, *J* = 2.0 Hz, 1H, H^{F3}), 8.32 (d, *J* = 1.9 Hz, 1H, H^{E3}), 7.96 (d, *J* = 8.1 Hz, 2H, H^{D3+B3}), 7.92 (m, 1H, H^{B4}), 7.88 (d, *J* = 5.9 Hz, 1H, H^{E6}), 7.83 (ddd, *J* = 8.2, 7.6, 1.5 Hz, 1H, H^{D4}), 7.78 (d, *J* = 2.0 Hz, 1H, H^{A3}), 7.75 (d, *J* = 5.7 Hz, 1H, H^{B6}), 7.54 (m, 2H, H^{H2}), 7.50 (m, 2H, H^{J2+J6}), 7.47–7.37 (m, 8H, $H^{C3+D6+E5+F5+H3+J3+J5}$), 7.33–7.27 (m, 2H, H^{H4+J4}), 7.15–7.09 (m, 3H, $H^{A5+B5+D5}$), 6.97 (tt, *J* = 7.6, 1.2 Hz, 1H, H^{G4}), 6.76 (broadened t, 2H, H^{G3}), 6.68 (dd, *J* = 8.0, 1.9 Hz, 1H, H^{C5}), 6.58 (br, 2H, H^{G2}), 6.11 (d, *J* = 8.0 Hz, 1H, H^{A6}), 5.72 (d, *J* = 7.9 Hz, 1H, H^{C6}), 1.49 (s, 9H, H^{tBu-F}), 1.44 (s, 9H, H^{tBu-E}). ^{13}C NMR (126 MHz, CD_2Cl_2) δ / ppm 169.2 (C^{B2}), 167.7 (C^{D2}), 166.1 (C^{F6}), 164.9 (C^{F4}), 164.5 (C^{E4}), 157.4 (C^{F2}), 157.3 (C^{E2}), 151.0 (C^{C1}), 150.4 (C^{E6}), 149.6 (C^{D6}), 149.5 (C^{B6}), 146.6 (C^{A1}), 144.2 (C^{A2}), 144.0 (C^{C2}), 142.2 (C^{J1}), 141.3 (C^{H1}), 138.9 (C^{G1}), 138.8 (C^{D4}), 138.7 (C^{B4}), 136.3 (C^{A4}), 134.2 (C^{C4}), 132.5 (C^{C6}), 131.4 (C^{A6}), 130.2 (C^{A5}), 129.4 (C^{H3}), 129.3 (C^{J3+J5}), 129.1 (C^{C5}), 128.9 (C^{G4}), 128.4 (C^{G3}), 128.2 (C^{G2}), 127.6 (C^{F5}), 127.5 (C^{H4}), 127.0 (C^{H2}), 126.9 (C^{J2+J6}), 125.8 (C^{E5}), 124.1 (C^{D5}), 123.7 (C^{C3}), 123.6 (C^{A3}), 123.2 (C^{B5}), 122.0 (C^{E3}), 120.8 (C^{F3}), 120.6 ($C^{B3/D3}$), 120.5 ($C^{B3/D3}$), 36.2 ($C^{quat-tBu-E/F}$), 36.1 ($C^{quat-tBu-E/F}$), 30.6 (C^{tBu-}

^{F}), 30.5 (C^{tBu-E}). IR (solid, $\tilde{\nu}$ / cm^{-1}) 2959 (w), 1610 (m), 1600 (m), 1563 (w), 1544 (w), 1477 (m), 1427 (m), 1387 (w), 1369 (w), 1252 (w), 1224 (w), 1166 (w), 1068 (w), 1030 (w), 908 (w), 876 (w), 840 (s), 824 (s), 785 (m), 758 (s), 718 (w), 696 (s), 638 (w), 609 (w), 598 (w), 584 (w), 557 (s), 523 (w), 484 (w). UV-Vis (CH_2Cl_2, 1.00 × 10^{-5} mol dm^{-3}) λ / nm (ε / dm^3 mol^{-1} cm^{-1}) 278 (97 000), 295 sh (71 000), 315 sh (37 000), 345 (15 000), 375 (7 000), 420 nm (5 000). Emission (CH_2Cl_2, 1.00 × 10^{-5} mol dm^{-3}, λ_{exc} = 420 nm) λ_{em} = 590, 639 sh nm. ESI-MS *m/z* 997.9 $[M–PF_6]^+$ (base peak, calc. 997.3). Found C 60.61, H 4.94, N 4.95; $C_{58}H_{52}F_6IrN_4P$ requires C 60.99, H 4.59, N 4.90%.

[Ir(Ph₂ppy)₂(1)][PF₆]

$[Ir_2(Ph_2ppy)_4Cl_2]$ (200 mg, 0.197 mmol) and **1** (31.1 mg, 0.199 mmol) were dissolved in MeOH (30 mL). An excess of solid NH_4PF_6 (10 eq) was added and the reaction mixture was stirred for 1 h at room temperature. The yellow precipitate that formed was separated by filtration, washed with MeOH and Et_2O and then purified by column chromatography (Fluka silica gel 60, CH_2Cl_2 changing to CH_2Cl_2:MeOH 100:0.5). After crystallization from a CH_2Cl_2 solution overlaid with EtOH : n-hexane (2:1), $[Ir(Ph_2ppy)_2(\mathbf{1})][PF_6]$ was obtained as an orange solid (142 mg 0.128 mmol, 65%). ^{1}H NMR (500 MHz, CD_2Cl_2) δ / ppm 8.27 (d, *J* = 8.1 Hz, 2H, H^{E3}), 8.03 (td, *J* = 7.9, 1.6 Hz, 2H, H^{E4}), 7.95-7.90 (m, 4H, H^{A3+E6}), 7.78 (m, 4H, H^{H2}), 7.53 (d, *J* = 8.3 Hz, 2H, H^{B3}), 7.51–7.45 (m, 6H^{H3+E5}), 7.38 (m, 2H, H^{H4}), 7.31 (m, 2H, H^{B4}), 7.21 (d, *J* = 2.1 Hz, 2H, H^{A5}), 6.95 (m, 2H, $H^{K2/K6}$), 6.85 (m, 2H, H^{B6}), 6.84-6.78 (m, 4H, H^{K3+K5}), 6.60 (m, 2H, H^{K4}), 6.50-6.44 (m, 4H, $H^{B5+K2/K6}$). ^{13}C NMR (126 MHz, CD_2Cl_2) δ / ppm 167.2 (C^{B2}), 156.0 (C^{E2}), 152.0 (C^{A6}), 150.2 (C^{E6}), 148.9 (C^{B6}), 147.7 (C^{A2}), 145.5 (C^{K1}), 144.3 (C^{A1}), 141.2 (C^{H1}), 140.3 (C^{E4}), 137.8 (C^{B4}), 135.9 (C^{A4}), 132.0 (C^{A5}), 129.5 (C^{H3}), 129.4 ($C^{K2/K6}$), 129.2 ($C^{K2/K6}$), 129.0 (C^{E5}), 127.7 (C^{H4}), 127.6 ($C^{K3/K5}$), 127.3 (C^{K4}), 127.1 (C^{H2}), 126.6 ($C^{K3/K5}$), 125.0 (C^{E3}), 122.9 (C^{A3}), 122.8 (C^{B5}), 120.5 (C^{B3}). IR (solid, $\tilde{\nu}$ / cm^{-1}) 3044 (w), 1600 (m), 1565 (w), 1480 (m), 1445 (w), 1408 (w), 1381 (w), 1345 (w), 1313 (w), 1295 (w), 1243 (w), 1177 (w), 1165 (w), 1103 (w), 1072 (w), 1031 (w), 1020 (w), 908 (w), 880 (w), 834 (s), 774 (m), 760 (s), 716 (m), 699 (s), 636 (m), 612 (w), 600 (m), 557 (s), 525 (m), 495 (w). UV-Vis (CH_2Cl_2, 1.00 × 10^{-5} mol dm^{-3}) λ / nm (ε / dm^3 mol^{-1} cm^{-1}) 255 (72 000), 282 (72 000), 299 (68 000), 325 (sh, 23 000), 345 (12 000), 400 (8 000), 420 nm (sh, 5 000 dm^3 mol^{-1} cm^{-1}). Emission (CH_2Cl_2, 1.00 × 10^{-5} mol dm^{-3}, λ_{exc} = 420 nm) λ_{em} = 611, 638 sh nm. ESI-MS *m/z* 961.8 $[M–PF_6]^+$ (base peak, calc. 961.2). Found C 60.50, H 3.88, N 5.17; $C_{56}H_{40}F_6IrN_4P$ requires C 60.81, H 3.64, N 5.07%.

[Ir(Ph₂ppy)₂(2)][PF₆]

The method was as for $[Ir(Ph_2ppy)_2(\mathbf{1})][PF_6]$ starting from $[Ir(Ph_2ppy)_4Cl_2]$ (200 mg, 0.197 mmol) and **2** (46.3 mg, 0.199 mmol). $[Ir(Ph_2ppy)_2(\mathbf{2})][PF_6]$ was isolated as an orange solid (152 mg, 0.129 mmol, 65%). ^{1}H NMR (500 MHz, CD_2Cl_2) δ / ppm 8.36 (m, 2H, H^{E3+F3}), 8.12–8.03 (overlapping m, 3H, $H^{E4+F4+E6}$), 7.68 (d, *J* = 5.7 Hz, 1H, H^{B6}), 7.65 (m, 2H, H^{H2}), 7.57 (m, 3H, H^{A3+J2}), 7.45 (m, 4H, H^{H3+J3}), 7.40–7.31 (overlapping m, 7H, $H^{B3+B4+D4+E5+F5+H4+J4}$), 7.18 (d, *J* = 8.1 Hz, 1H, H^{D3}), 7.15 (dd, *J* = 5.9, 0.7 Hz, 1H, H^{D6}), 7.06 (d, *J* = 2.1 Hz, 1H, H^{C3}), 6.98 (m,

1H, H^{G4}), 6.92 (m, 3H, H^{G3+L6}), 6.88 (d, J = 2.1 Hz, 1H, H^{A5}), 6.84 (m, 3H, H^{G2+L4}), 6.79–6.71 (overlapping m, 3H, $H^{B5+K4+L3}$), 6.67–6.58 (overlapping m, 3H, $H^{D5+K3+L5}$), 6.56 (d, J = 2.1 Hz, 1H, H^{C5}), 6.52 (m, 2H, H^{K5+K6}), 6.05 (dt, J = 7.5, 1.3 Hz, 1H, H^{L2}), 5.98 (m, 1H, H^{K2}). ^{13}C NMR (126 MHz, CD_2Cl_2) δ / ppm 169.3 (C^{D2}), 166.7 (C^{B2}), 165.2 (C^{F6}), 157.8 (C^{E2}), 157.1 (C^{F2}), 151.2 (C^{A6}), 150.5 (C^{C6}), 149.9 (C^{E4}), 149.5 (C^{B6}), 148.3 (C^{D6}), 147.7 (C^{C2}), 147.3 (C^{A2}), 146.0 (C^{L1}), 144.4 (C^{K1}), 141.9 (C^{J1}), 141.4 (C^{C1}), 140.9 (C^{H1}), 140.3 (C^{F4}), 140.1 (C^{E6}), 137.9 (C^{B4}), 137.6 (C^{G1}), 137.5 (C^{D4}), 135.7 (C^{A4}), 134.4 (C^{A1}), 134.1 (C^{C4}), 132.4 (C^{A5}), 131.7 (C^{C5}), 131.2 (C^{F5}), 130.6 (C^{L2}), 129.7 (C^{K6}), 129.5 (C^{K2}), 129.5 (C^{G4}), 129.4 ($C^{H3/J3}$), 129.3 ($C^{H3/J3}$), 129.0 (C^{G2}), 128.3 (C^{L6}), 128.3 (C^{E5}), 128.3 (C^{G3}), 127.7 (C^{H4}), 127.2 (C^{J4}), 127.0 ($C^{H2/J2}$), 127.0 ($C^{H2/J2}$), 127.0 (C^{K3+L3}), 126.5 (C^{K4}), 126.5 (C^{K5}), 126.4 (C^{L5}), 126.1 (C^{L4}), 125.8 (C^{E3}), 125.0 (C^{F3}), 123.6 (C^{C3}), 123.2 (C^{B5}), 122.9 (C^{A3}), 122.1 (C^{D5}), 120.8 (C^{D3}), 120.7 (C^{B3}). IR (solid, $\tilde{\nu}$ / cm^{-1}) 3031 (w), 1598 (m), 1563 (w), 1481 (m), 1444 (m), 1411 (w), 1379 (w), 1346 (w), 1294 (w), 1233 (w), 1170 (w), 1071 (w), 1026 (w), 996 (w), 905 (w), 887 (w), 834 (s), 787 (m), 757 (s), 717 (w), 702 (s), 693 (s), 630 (m), 599 (m), 555 (s), 526 (m), 506 (w), 488 (w). UV-Vis (CH_2Cl_2, 1.00 × 10^{-5} mol dm^{-3}) λ / nm (ε / dm^3 mol^{-1} cm^{-1}) 261 (70 000), 280 (69 000), 294 (67 000), 325 sh (23 000), 345 (14 000), 400 (6 000), 420 sh (4 000). Emission (CH_2Cl_2, 1.00 × 10^{-5} mol dm^{-3}, λ_{exc} = 420 nm) λ_{em} = 615 sh, 645 nm. ESI-MS *m/z* 1037.8 [M–PF_6]$^+$ (base peak, calc. 1037.3). Found C 61.92, H 4.02, N 4.75; $C_{62}H_{44}F_6IrN_4P{\cdot}H_2O$ requires C 62.04, H 3.86, N 4.67%.

[Ir(Ph_2ppy)$_2$(3)][PF_6]

The method was as for [Ir(Ph_2ppy)$_2$(**1**)][PF_6] starting from [Ir(Ph_2ppy)$_4$$Cl_2$] (200 mg, 0.197 mmol) and **3** (53.5 mg, 0.199 mmol) in MeOH (10 mL). [Ir(Ph_2ppy)$_2$(**3**)][PF_6] was isolated as an orange solid (131 mg, 0.108 mmol, 55%). ^{1}H NMR (500 MHz, CD_2Cl_2) δ / ppm 8.01 (d, J = 1.9 Hz, 2H, H^{E3}), 7.94 (d, J = 2.1 Hz, 2H, H^{A3}), 7.82–7.74 (m, 6H, H^{H2+E6}), 7.53 (d, J = 8.3 Hz, 2H, H^{B3}), 7.50 (m, 4H, H^{H3}), 7.44 (dd, J = 6.0, 1.9 Hz, 2H, H^{E5}), 7.39 (m, 2H, H^{H4}), 7.32 (ddd, J = 8.4, 7.4, 1.5 Hz, 2H, H^{B4}), 7.22 (d, J = 2.1 Hz, 2H, H^{A5}), 6.99 (m, 2H, $H^{K2/K6}$), 6.85 (ddd, J = 5.9, 1.4, 0.7 Hz, 2H, H^{B6}), 6.82 (m, 4H, H^{K3+K5}), 6.59 (m, 2H, H^{K4}), 6.50–6.45 (m, 4H, $H^{B5+K2/K6}$), 1.34 (s, 18H, H^{tBu}).^{13}C NMR (126 MHz, CD_2Cl_2) δ / ppm 167.3 (C^{B2}), 164.9 (C^{E4}), 155.8 (C^{E2}), 152.0 (C^{A6}), 149.8 (C^{E6}) , 149.0 (C^{B6}), 147.7 (C^{A2}), 145.6 (C^{K1}), 145.0 (C^{A1}), 141.2 (C^{H1}), 137.7 (C^{B4}), 135.8 (C^{A4}), 132.0 (C^{A5}), 129.5 (C^{H3}), 129.4 ($C^{K2/K6}$), 129.1 ($C^{K2/K6}$), 127.7 ($C^{H4+K3/K5}$), 127.4 (C^{K4}), 127.1 (C^{H2}), 126.5 ($C^{K3/K5}$), 126.2 (C^{E5}), 122.8 (C^{A3}), 122.6 (C^{B5}), 121.2 (C^{E3}), 120.4 (C^{B3}), 36.1 ($C^{quat\text{-}tBu}$), 30.5 (C^{tBu}). IR (solid, $\tilde{\nu}$ / cm^{-1}) 2965 (w), 1609 (w), 1567 (w), 1541 (w), 1481 (m), 1411 (m), 1382 (w), 1296 (w), 1246 (w), 1169 (w), 1155 (w), 1070 (w), 1019 (w), 895 (w), 878 (w), 835 (s), 782 (m), 772 (m), 758 (s), 737 (w), 704 (s), 637 (w), 610 (w), 599 (w), 556 (s), 525 (w), 498 (w). UV-Vis (CH_2Cl_2, 1.00 × 10^{-5} mol dm^{-3}) λ / nm (ε / dm^3 mol^{-1} cm^{-1}) 256 (79 000), 280 (79 000), 300 (71 000), 325 sh (27 000), 345 (14 000), 400 (8 000), 420 sh (5 000). Emission (CH_2Cl_2, 1.00 × 10^{-5} mol dm^{-3}, λ_{exc} = 420 nm) λ_{em} = 588, 639 sh nm. ESI-MS *m/z* 1073.9 [M–PF_6]$^+$ (base peak, calc. 1073.4). Found C 62.96, H 4.80, N 4.88; $C_{64}H_{56}F_6IrN_4P$ requires C 63.09, H 4.63, N 4.60%.

[Ir(Ph$_2$ppy)$_2$(4)][PF$_6$]

The method was as for [Ir(Ph$_2$ppy)$_2$(**1**)][PF$_6$] starting from [Ir(Ph$_2$ppy)$_4$Cl$_2$] (150 mg, 0.148 mmol) and **4** (51.5 mg, 0.149 mmol). [Ir(Ph$_2$ppy)$_2$(**4**)][PF$_6$] was isolated as a yellow solid (106 mg, 0.082 mmol, 55%). ^{1}H NMR (500 MHz, CD$_2$Cl$_2$) δ / ppm 8.12 (d, J = 1.8 Hz, 1H, H^{E3}), 8.11 (d, J = 2.0 Hz, 1H, H^{F3}), 7.96 (d, J = 6.0 Hz, 1H, H^{E6}), 7.67–7.62 (m, 3H, H^{B6+H2}), 7.58 (d, J = 2.1 Hz, 1H, H^{A3}), 7.58–7.55 (m, 2H, H^{J2}), 7.44 (m, 4H, H^{H3+J3}), 7.38–7.31 (overlapping m, 6H, H$^{B3+B4+D4+E5+H4+J4}$), 7.28 (d, J = 2.0 Hz, 1H, H^{F5}), 7.19 (d, J = 8.1 Hz, 1H, H^{D3}), 7.14 (dd, J = 5.9, 0.7 Hz, 1H, H^{D6}), 7.07 (d, J = 2.1 Hz, 1H, H^{C3}), 6.98 (m, 1H, H^{G4}), 6.93 (m, 2H, H^{G3}), 6.89 (m, 1H, H^{L6}), 6.87 (d, J = 2.1 Hz, 1H, H^{A5}), 6.84 (m, 3H, H^{G2+L4}), 6.78–6.71 (overlapping m, 3H, H$^{B5+K4+L3}$), 6.67–6.58 (overlapping m, 3H, H$^{D5+K3+L5}$), 6.55 (d, J = 2.1 Hz, 1H, H^{C5}), 6.53 (m, 2H, H^{K5+K6}), 6.05 (dt, J = 7.5, 1.3 Hz, 1H, H^{L2}), 5.98 (m, 1H, H^{K2}), 1.39 (s, 9H, H^{tBu-F}), 1.37 (s, 9H, H^{tBu-E}). ^{13}C NMR (126 MHz, CD$_2$Cl$_2$) δ / ppm 169.4 (C^{D2}), 166.8 (C^{B2}), 165.1 (C^{F6}), 164.8 (C^{E4+F4}), 157.7 (C^{E2}), 157.3 (C^{F2}), 151.3 (C^{A6}), 150.5 (C^{C6}), 149.5 (C^{E6}), 149.5 (C^{B6}), 148.4 (C^{D6}), 147.7 (C^{C2}), 147.3 (C^{A2}), 146.0 (C^{L1}), 144.4 (C^{K1}), 141.9 (C^{J1}), 141.7 (C^{C1}), 141.0 (C^{H1}), 138.0 (C^{G1}), 137.7 (C^{B4}), 137.4 (C^{D4}), 135.6 (C^{A4}), 134.9 (C^{A1}), 134.0 (C^{C4}), 132.3 (C^{A5}), 131.6 (C^{C5}), 130.6 (C^{L2}), 129.6 (C^{K6}), 129.5 (C^{K2}), 129.4 (C^{H3}), 129.4 (C^{G4}), 129.3 (C^{J3}), 129.0 (C^{G2}), 128.4 (C^{L6}), 128.3 (C^{G3}), 128.1 (C^{F5}), 127.7 (C^{H4}), 127.1 (C^{J4}), 127.0 (C^{H2+J2}), 127.0 (C^{K3}), 126.9 (C^{L3}), 126.5 (C^{K5}), 126.4 (C^{K4}), 126.4 (C^{L5}), 126.1 (C^{L4}), 125.6 (C^{E5}), 123.5 (C^{C3}), 123.0 (C^{B5}), 122.8 (C^{A3}), 122.0 (C^{E3}), 121.9 (C^{D5}), 121.7 (C^{F3}), 120.8 (C^{D3}), 120.7 (C^{B3}), 36.1 (C$^{quat-tBu-E}$), 36.0 (C$^{quat-tBu-F}$), 30.5 (C^{tBu-F}), 30.4 (C^{tBu-E}). IR (solid, $\tilde{\nu}$ / cm^{-1}) 3596 (w), 3031 (w), 1598 (m), 1563 (w), 1481 (m), 1444 (m), 1411 (w), 1379 (w), 1346 (w), 1294 (w), 1233 (w), 1170 (w), 1071 (w), 1026 (w), 996 (w), 905 (w), 887 (w), 834 (s), 787 (m), 757 (s), 717 (w), 702 (s), 693 (s), 630 (m), 599 (m), 555 (s), 526 (m), 506 (w), 488 (w). UV-Vis (CH$_2$Cl$_2$, 1.00 × 10^{-5} mol dm^{-3}) λ / nm (ε / dm^3 mol^{-1} cm^{-1}) 261 (74 000), 280 (72 000), 298 (69 000), 325 sh (29 000), 345 (14 000), 400 (6 000), 420 nm (4 000). Emission (CH$_2$Cl$_2$, × 10^{-5} mol dm^{-3}, λ$_{exc}$ = 420 nm) λ$_{em}$ = 609, 636 sh nm. ESI-MS m/z 1149.9 [M–PF$_6$]$^+$ (base peak, calc. 1149.5). Found C 64.54, H 4.88, N 4.61; $C_{70}H_{60}F_6IrN_4P\cdot 0.5H_2O$ requires C 64.50, H 4.72, N 4.30%.

Crystallography

Single crystal data were collected on a Bruker APEX-II diffractometer with data reduction, solution and refinement using the programs APEX[3] and CRYSTALS[4] or SHELX-13.[5] ORTEP diagrams and structure analysis used Mercury v. 3.0.1 and v. 3.3.[6,7]

HPh$_2$ppy

$C_{23}H_{17}N$, M = 307.38, colourless block, orthorhombic, space group $Pna2_1$, a = 7.4701(2), b = 19.7919(5), c = 11.1894(3) Å, U = 1654.32(8) Å^3, Z = 4, D_c = 1.234 Mg m^{-3}, μ(Cu-Kα) = 0.544 mm^{-1}, T = 296 K. Total 11157 reflections, 2874 unique, R_{int} = 0.0255. Refinement of 2734 reflections (217 parameters) with I >2σ (I) converged at final $R1$ = 0.0343 ($R1$ all data = 0.0360), $wR2$ = 0.0966 ($wR2$ all data = 0.0986), gof = 1.052. CCDC 1019226.

[Ir(Phppy)$_2$(1)][PF$_6$]

$C_{44}H_{32}F_6IrN_4P$, *M* = 953.93, orange block, monoclinic, space group *P*2$_1$/*n*, *a* = 15.0877(10), *b* = 13.1747(9), *c* = 18.0930(12) Å, β = 96.094(2)°, *U* = 3576.1(4) Å^3, *Z* = 4, D_c = 1.772 Mg m^{-3}, μ(Cu-Kα) = 8.270 mm^{-1}, *T* = 123 K. Total 63369 reflections, 6470 unique, R_{int} = 0.0329. Refinement of 6256 reflections (505 parameters) with $I > 2\sigma(I)$ converged at final *R*1 = 0.0192 (*R*1 all data = 0.0199), *wR*2 = 0.0487 (*wR*2 all data = 0.0492), gof = 1.054. CCDC 1019228.

[Ir(Ph$_2$ppy)$_2$(1)][PF$_6$]·EtOH

$C_{58}H_{46}F_6IrN_4OP$, *M* = 1152.18, orange block, orthorhombic, space group *Pna*2$_1$, *a* = 13.1280(6), *b* = 37.8916(17), *c* = 10.2900(5) Å, *U* = 5118.7(4) Å^3, *Z* = 4, D_c = 1.495 Mg m^{-3}, μ(Cu-Kα) = 5.901 mm^{-1}, *T* = 123 K. Total 48035 reflections, 9189 unique, R_{int} = 0.0435. Refinement of 8655 reflections (670 parameters) with $I > 2\sigma(I)$ converged at final *R*1 = 0.0467 (*R*1 all data = 0.0493), *wR*2 = 0.1210 (*wR*2 all data = 0.1228), gof = 1.117. CCDC 1019227.

[Ir(Ph$_2$ppy)$_2$(2)][PF$_6$]·2C$_6$H$_5$Me

$C_{76}H_{60}F_6IrN_4P$, *M* = 1366.52, yellow block, triclinic, space group *P*–1, *a* = 10.8073(6), *b* = 13.5065(8), *c* = 20.8777(12) Å, α = 80.953(3), β = 86.577(3), γ = 78.797(3)°, *U* = 2950.90(17) Å^3, *Z* = 2, D_c = 1.538 Mg m^{-3}, μ(Cu-Kα) = 5.207 mm^{-1}, *T* = 123 K. Total 50598 reflections, 10694 unique, R_{int} = 0.037. Refinement of 10647 reflections (793 parameters) with $I > 2\sigma(I)$ converged at final *R*1 = 0.0204 (*R*1 all data = 0.0243), *wR*2 = 0.0474 (*wR*2 all data = 0.0492), gof = 0.9016. CCDC 1019229.

Device preparation

Glass substrates partially coated with indium-tin-oxide (ITO) (www.naranjosubstrates.com) were cleaned by 5 mins sonication in soapy water, deionized water, isopropanol and thereafter left under an UV-O_3 lamp for 20 minutes. A 60 nm thick PEDOT/PSS film was spincoated on top of the glass substrate at 1000 rpm, and then dried at 150ºC for 15 minutes. On top of this a blend of the iridium complex and the ionic liquid [BMIM][PF$_6$] (1-butyl-3-methyl-imidazolium hexafluoridophosphate) (molar ratio 4:1) was spin coated from an acetonitrile solution (20 mg/ml) at 1000 rpm, leading to a 100 nm thick active layer. Finally 70 nm of aluminum thermally evaporated on top of the device to serve as the cathode contact

Crystal structure of HPh_2ppy

Although the synthesis of HPh_2ppy has been described, the crystal structure of the compound has not been reported. Single crystals of HPh_2ppy were grown from a CH_2Cl_2 solution of the compound layered with n-hexane and Fig. S1 depicts the molecular structure. Bond distances and angles are as expected, as is the twisted arrangement of the rings containing N1, C12 and C18 with respect to the central arene ring (angles between the planes of the rings containing C6/N1, C6/C12 and C6/C18 are 32.2, 39.1 and 31.1°). HPh_2ppy crystallizes in the orthorhombic space group $Pna2_1$ and is isostructural with 1,3,5-triphenylbenzene,[8,9] indicating that exchange of one phenyl for a 2-pyridyl group has little influence on the molecular packing. However, on going to 1,3,5-tris(2-pyridyl)benzene (which also crystallizes in the space group $Pna2_1$),[10] the inter-ring twist angles are smaller (16.0–24.6°) than in HPh_2ppy and and 1,3,5-triphenylbenzene and the molecular packing is modified, although Williams and coworkers report the CH...N contacts (the factor that might be expected to be responible for the modification) to be very weak.[10]

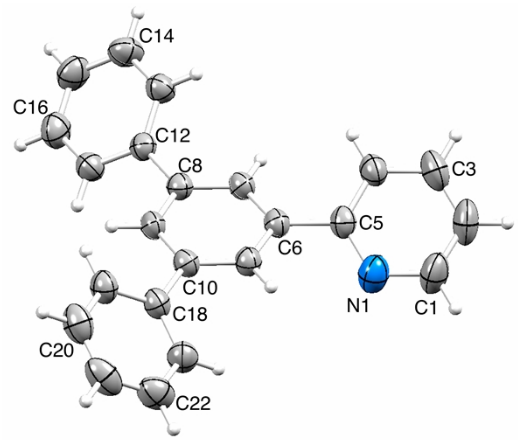

Fig. S1. Structure of HPh_2ppy with ellipsoids plotted at 40% probability level. Selected bond distances: N1–C5 = 1.364(3), N1–C1 = 1.366(3), C5–C6 = 1.485(3), C8–C12 = 1.489(3), C10–C18 = 1.488(3) Å.

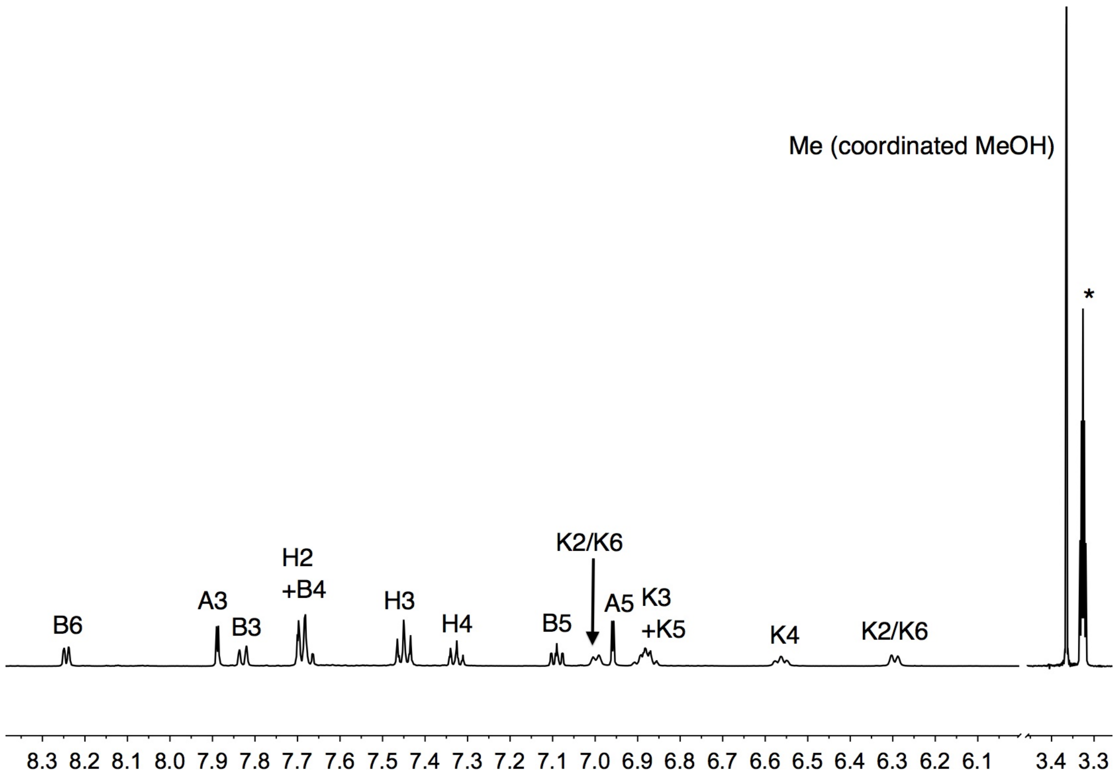

Fig. S2. 500 MHz ^{1}H NMR spectrum (295 K) of $[Ir(Ph_2ppy)_2(MeOH)_2][PF_6]$ in CD_3OD. * = residual solvent peak. Chemical shifts in δ / ppm.

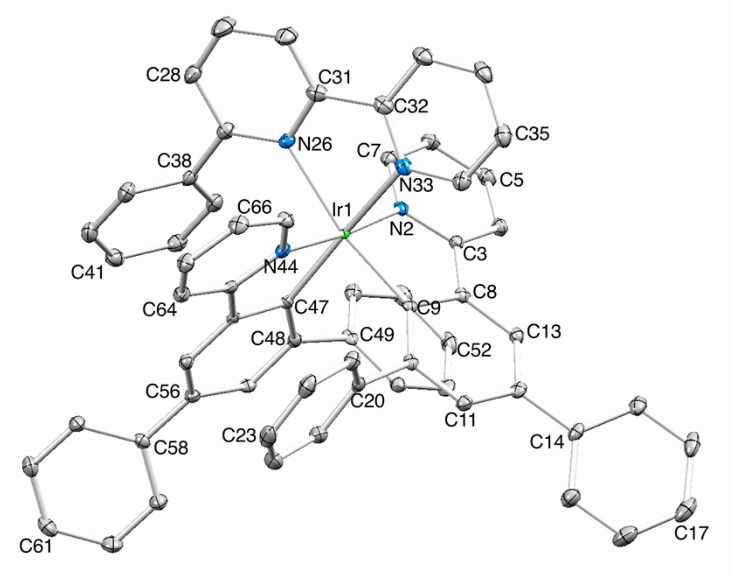

Fig. S3. The structure of the Λ-$[Ir(Ph_2ppy)_2(\mathbf{2})]^+$ cation in racemic $[Ir(Ph_2ppy)_2(\mathbf{2})][PF_6]\cdot 2C_6H_5Me$ (H atoms omitted, ellipsoids plotted at 40% probability). Selected bond metrics: Ir1–N2 = 2.0479(18), Ir1–C9 = 2.031(2), Ir1–N26 = 2.2610(18), Ir1–N33 = 2.1454(19), Ir1–N44 = 2.0549(18), Ir1–C47 = 2.047(2) Å; N26–Ir1–N33 = 76.14(7), N2–Ir1–C9 = 80.64(8), N44–Ir1–C47 = 80.11(8)°.

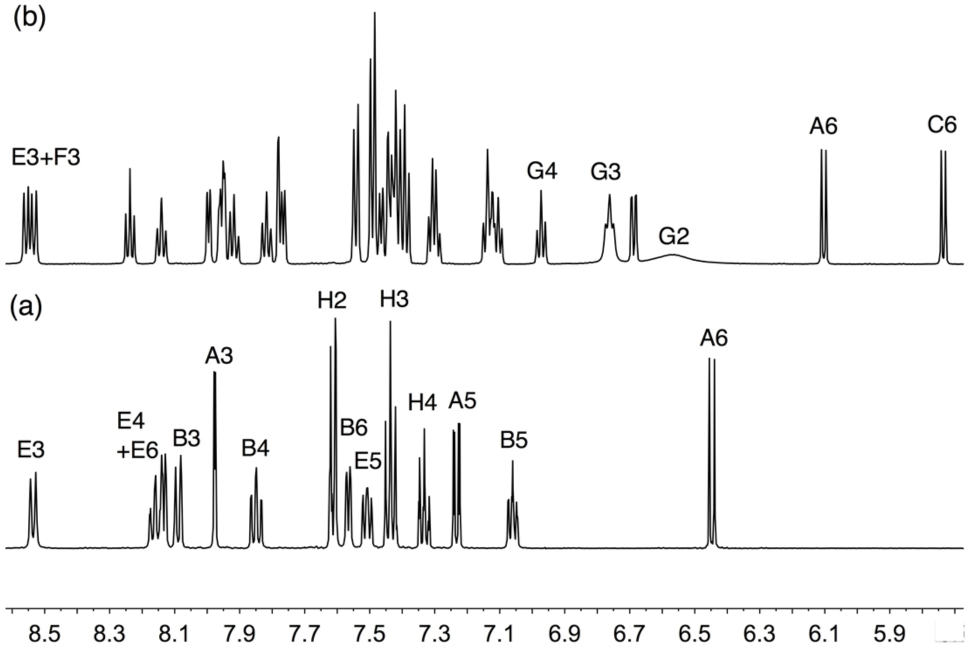

Fig. S4. ^{1}H NMR spectra of (a) [Ir(Phppy)$_2$(**1**)][PF$_6$] (500 MHz, 298 K, CD_2Cl_2) and (b) [Ir(Phppy)$_2$(**2**)][PF$_6$] (600 MHz, 298 K, CD_2Cl_2). See Scheme 2 for atom labelling. Chemical shifts in δ / ppm.

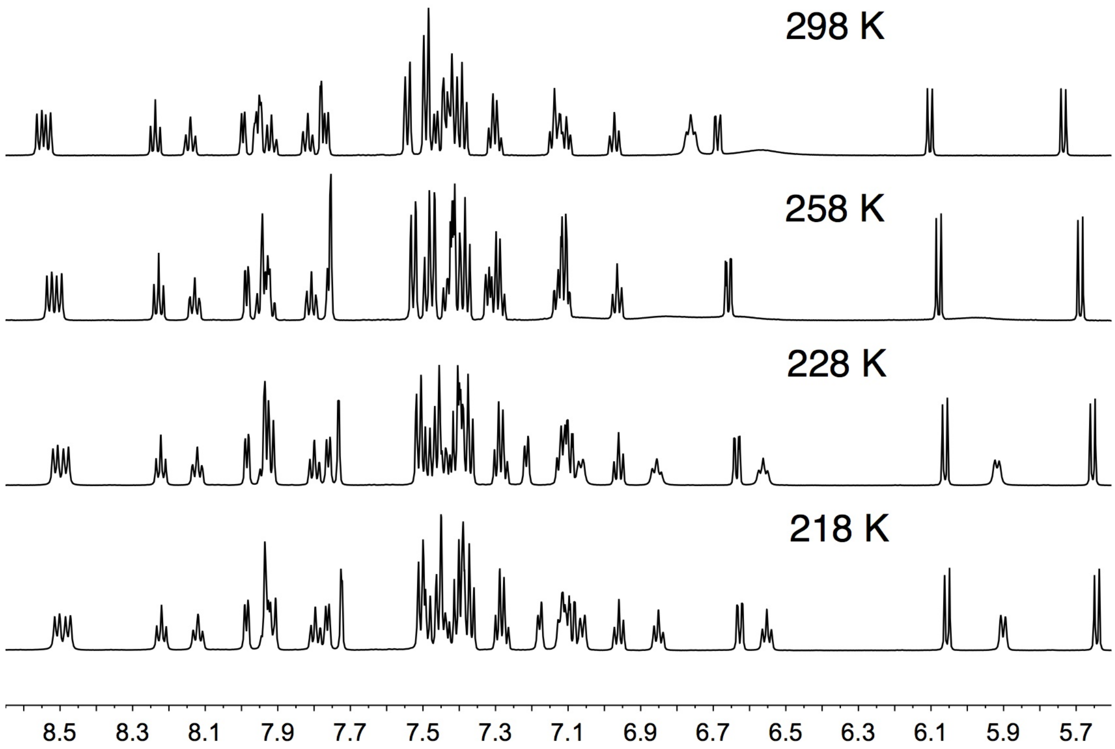

Fig. S5. The 600 MHz variable temperature ^{1}H NMR spectra of a CD_2Cl_2 solution of [Ir(Phppy)$_2$(**2**)][PF$_6$]. An expanded region is shown in Fig. 5a in the main paper.

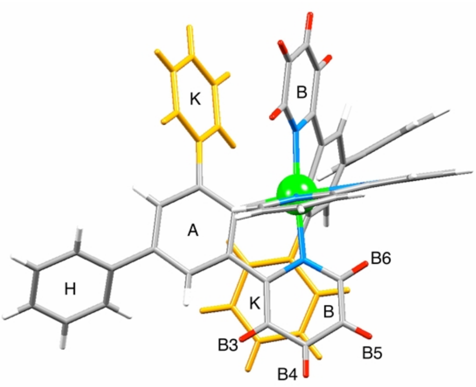

Fig. S6. π-Stacking of pyridine ring B and pendant phenyl ring K in the $[Ir(Ph_2ppy)_2(\mathbf{1})]^+$ cation which is consistent with the low-frequency ^1H NMR spectroscopic signals in Table S1. The diagram is the crystallographically determined structure.

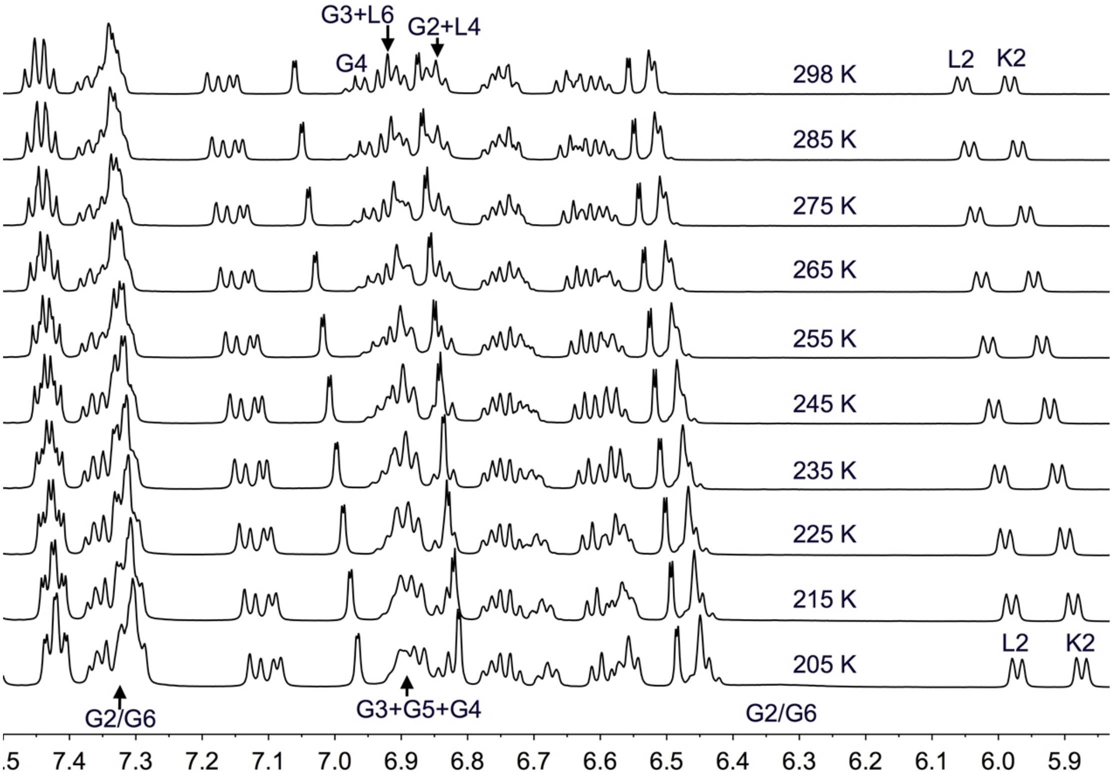

Fig. S7. Part of the 500 MHz variable temperature ^1H NMR spectra of a CD_2Cl_2 solution of $[Ir(Ph_2ppy)_2(\mathbf{2})][PF_6]$. Changes in signal integrals on cooling are consistent with the collapse of signals at δ 6.92 ppm (H^{G3}) and δ 6.84 ppm (H^{G2}). At 205 K, a broad peak (FWHM ≈ 70 Hz) appears at δ 6.33 ppm and is attributed to either H^{G2} or H^{G6}. Further cooling was not possible with available solvents. We propose that the distortion in the coordination sphere of the iridium atom caused by the π-stacking of rings B and L, and of D and K (and observed in the solid-state structure of $[Ir(Ph_2ppy)_2(\mathbf{2})][PF_6]$) is responsible for the less hindered rotation of ring G.

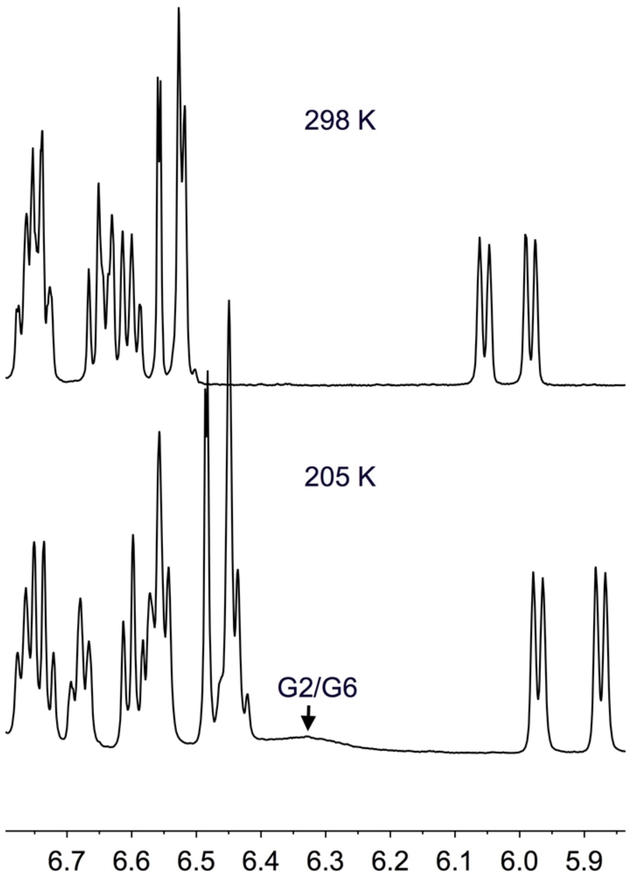

Fig. S8. Expansion of the 500 MHz ^{1}H NMR spectrum at 205 K of a CD_2Cl_2 solution of $[Ir(Ph_2ppy)_2(\mathbf{2})][PF_6]$, showing the broad signal (FWHM ≈ 70 Hz) assigned to H^{G2} or H^{G6}.

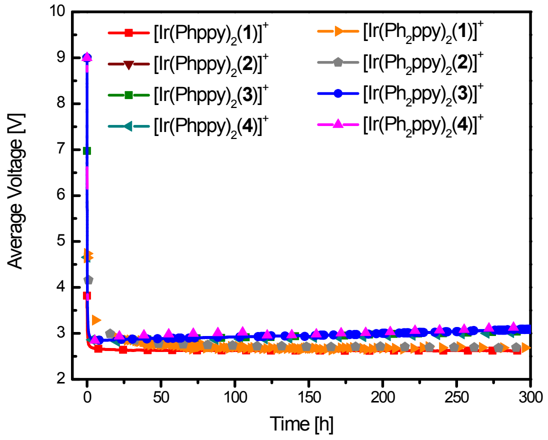

(a)

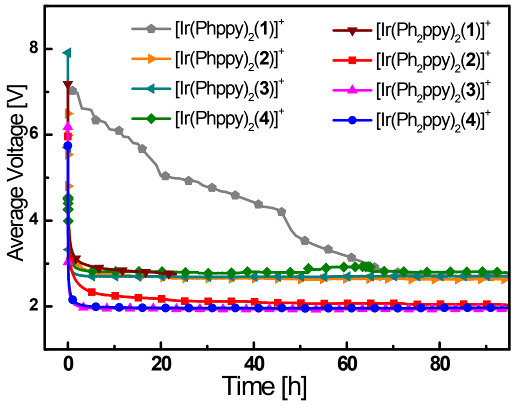

(b)

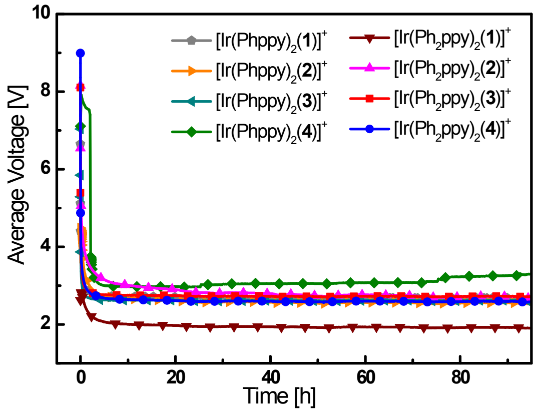

(c)

Fig. S9. Voltage vs time (a) at 300 A/m^2, (b) at 100 A/m^2 and (c) at 50 A/m^2, at a frequency of 1 kHz and duty cycles of 50% for all devices.

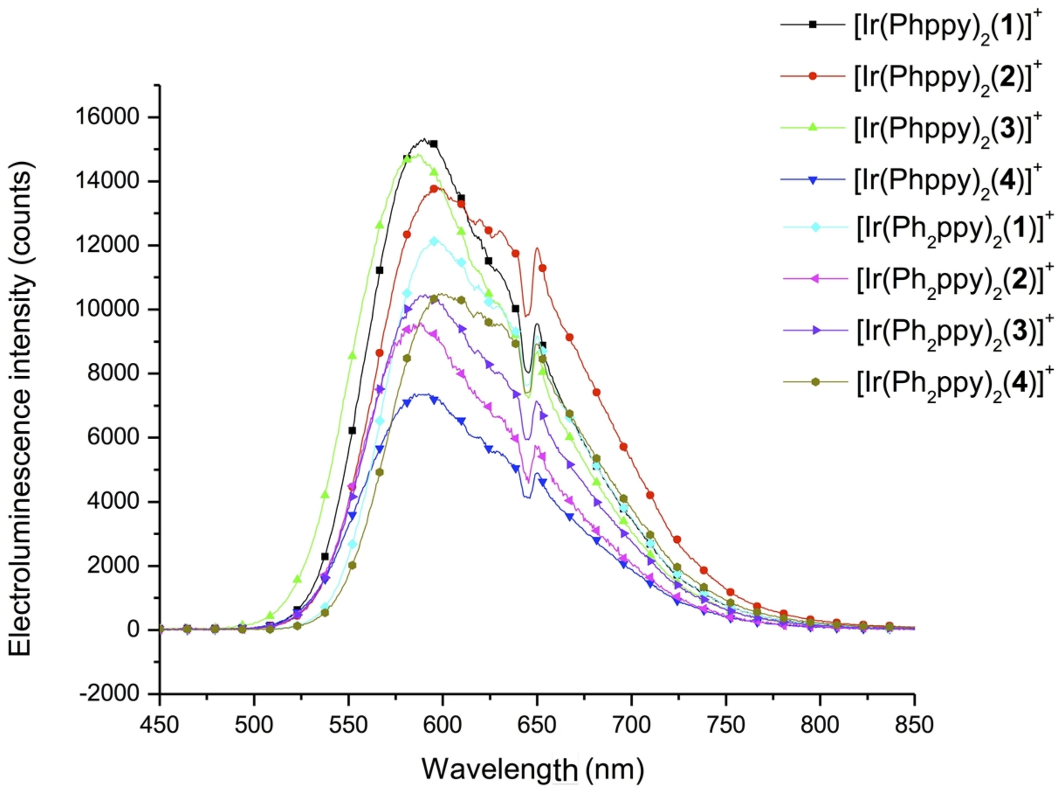

Fig. S10. Electroluminescence of the iridium compounds, measured at 50 A/m^2 at a frequency of 1 kHz and duty cycles of 50% for all devices.

Table S1. ^1H NMR spectrscopic data (CD_2Cl_2, 298 K) for rings B and K in the C_2-symmetric complexes $[Ir(Phppy)_2(N^\wedge N)][PF_6]$ and $[Ir(Ph_2ppy)_2(N^\wedge N)][PF_6]$ (N^N = **1** or **3**).

Cation	H^{B3}	H^{B4}	H^{B5}	H^{B6}
$[Ir(Phppy)_2(\mathbf{1})]^+$	8.09	7.85	7.06	7.56
$[Ir(Phppy)_2(\mathbf{3})]^+$	8.09	7.86	7.08	7.58
$[Ir(Ph_2ppy)_2(\mathbf{1})]^+$	7.53	7.31	6.48[a]	6.85
$[Ir(Ph_2ppy)_2(\mathbf{3})]^+$	7.53	7.32	6.48[a]	6.85
	H^{K2}, H^{K6}	H^{K3} + H^{K5}	H^{K4}	
$[Ir(Ph_2ppy)_2(\mathbf{1})]^+$	6.95, 6.45[a]	6.82	6.60	
$[Ir(Ph_2ppy)_2(\mathbf{3})]^+$	6.99, 6.47[a]	6.82	6.59	

[a] Values determined from HMQC spectra

1 P. G. Bomben, B. D. Kolvisto and C. P. Berlinguette, *Inorg. Chem.*, 2010, **49**, 4960.

2 J. D. Kehlbeck, E. J. Dimise, S. M. Sparks, S. Ferrara, J. M. Tanski and C. M. Anderson, *Synthesis*, 2007, 1979.

3 Bruker Analytical X-ray Systems, Inc., 2006, APEX2, version 2 User Manual, M86-E01078, Madison, WI.

4 P. W. Betteridge, J. R. Carruthers, R. I. Cooper, K. Prout and D. J. Watkin, *J. Appl. Cryst.*, 2003, **36**, 1487.

5 G. M. Sheldrick, *Acta Crystallogr., Sect. A*, 2008, **64**, 112.

6 I. J. Bruno, J. C. Cole, P. R. Edgington, M. K. Kessler, C. F. Macrae, P. McCabe, J. Pearson and R. Taylor, *Acta Crystallogr., Sect. B* 2002, **58**, 389.

7 C. F. Macrae, I. J. Bruno, J. A. Chisholm, P. R. Edgington, P. McCabe, E. Pidcock, L. Rodriguez-Monge, R. Taylor, J. van de Streek and P. A. Wood, *J. Appl. Cryst.*, 2008, **41**, 466.

8 Y. C. Lin and D. E. Williams, *Acta Crystallogr., Sect. B*, 1975, **31**, 318.

9 D. Prasad, A. Preetam and M. Nath, *Compt. Rend. Chim.*, 2013, **16**, 252.

10 S. J. Farley, D. L. Rochester, A. L. Thompson, J. A. K. Howard and J. G. Williams, *Inorg. Chem.*, 2005, **44**, 9690.
